# Efficacy and Safety of Rivaroxaban for Postoperative Thromboprophylaxis in Patients After Bariatric Surgery: A Randomized Clinical Trial

**DOI:** 10.1001/jamanetworkopen.2023.15241

**Published:** 2023-05-25

**Authors:** Dino Kröll, Philipp C. Nett, Nikki Rommers, Yves Borbély, Fabian Deichsel, Antonio Nocito, Jörg Zehetner, Ulf Kessler, Yannick Fringeli, Lorenzo Alberio, Daniel Candinas, Guido Stirnimann

**Affiliations:** 1 Department of Visceral Surgery and Medicine, Bern University Hospital (Inselspital) and University of Bern, Bern, Switzerland; 2 Department of Clinical Research, Clinical Trial Unit, University of Basel, Switzerland; 3 Department of Surgery, Cantonal Hospital of Baden, Baden, Switzerland; 4 Department of Visceral Surgery, Hirslanden Clinic Beau-Site, Bern, Switzerland; 5 Division of Haematology and Central Haematology Laboratory, Centre Hospitalier Universitaire Vaudois, University of Lausanne, Lausanne, Switzerland

## Abstract

**Importance:**

Venous thromboembolism (VTE) is a leading cause of morbidity and mortality after bariatric surgery. Clinical end point studies on thromboprophylaxis with direct oral anticoagulants in patients undergoing bariatric surgery are lacking.

**Objective:**

To assess the efficacy and safety of a prophylactic dose of 10 mg/d of rivaroxaban for both 7 and 28 days after bariatric surgery.

**Design, Setting, and Participants:**

This assessor-blinded, phase 2, multicenter randomized clinical trial was conducted from July 1, 2018, through June 30, 2021, with participants from 3 academic and nonacademic hospitals in Switzerland.

**Intervention:**

Patients were randomized 1 day after bariatric surgery to 10 mg of oral rivaroxaban for either 7 days (short prophylaxis) or 28 days (long prophylaxis).

**Main Outcomes and Measures:**

The primary efficacy outcome was the composite of deep vein thrombosis (symptomatic or asymptomatic) and pulmonary embolism within 28 days after bariatric surgery. Main safety outcomes included major bleeding, clinically relevant nonmajor bleeding, and mortality.

**Results:**

Of 300 patients, 272 (mean [SD] age, 40.0 [12.1] years; 216 women [80.3%]; mean body mass index, 42.2) were randomized; 134 received a 7-day and 135 a 28-day VTE prophylaxis course with rivaroxaban. Only 1 thromboembolic event (0.4%) occurred (asymptomatic thrombosis in a patient undergoing sleeve gastrectomy with extended prophylaxis). Major or clinically relevant nonmajor bleeding events were observed in 5 patients (1.9%): 2 in the short prophylaxis group and 3 in the long prophylaxis group. Clinically nonsignificant bleeding events were observed in 10 patients (3.7%): 3 in the short prophylaxis arm and 7 in the long prophylaxis arm.

**Conclusions and Relevance:**

In this randomized clinical trial, once-daily VTE prophylaxis with 10 mg of rivaroxaban was effective and safe in the early postoperative phase after bariatric surgery in both the short and long prophylaxis groups.

**Trial Registration:**

ClinicalTrials.gov Identifier: NCT03522259

## Key Points

**Question:**

Is thromboprophylaxis with 10 mg of rivaroxaban for 7 and 28 days after bariatric surgery efficacious and safe?

**Findings:**

In this randomized clinical trial, 272 patients undergoing bariatric surgery were assigned to 10 mg of rivaroxaban for 7 or 28 days. Overall, a single asymptomatic thromboembolic event (0.4%) occurred in a patient undergoing sleeve gastrectomy in the long prophylaxis group, and major bleeding and clinically relevant nonmajor bleeding events occurred in 5 patients (1.9%).

**Meaning:**

This trial found that thromboembolic prophylaxis with 10 mg of rivaroxaban was efficacious and safe after bariatric surgery.

## Teaser

This randomized clinical trial assesses the efficacy and safety of rivaroxaban as postoperative thromboprophylaxis in patients undergoing bariatric surgery.

## Introduction

The epidemic increase in severe obesity and the effectiveness and safety of bariatric interventions (regarding body weight reduction and resolution of associated comorbidities as well as low postoperative complication rates) have led to a worldwide increase in the rate of bariatric surgery during the last few decades.^1,2,3,4^ Severe obesity^5,6^ and bariatric surgery^7,8,9^ are known risk factors for the development of venous thromboembolism (VTE). Deep vein thrombosis (DVT) and pulmonary embolism (PE), although not common complications (incidences between 0.1% and 3%),^9,10^ are characterized by a highly variable clinical presentation, ranging from asymptomatic incidental findings to life-threatening events. Venous thromboembolism is associated with significant morbidity and mortality after bariatric surgery, and most events (>70%) occur after hospital discharge within the first 30 days after surgery.^11^

All patients undergoing bariatric surgery are considered to be at least at moderate risk for VTE events because of severe obesity with associated comorbidities, laparoscopic surgery, and perioperative immobility.^12,13^ Clinical guidelines recommend some form of pharmacologic thromboprophylaxis after bariatric surgery in addition to mechanical prophylaxis to reduce the risk of thromboembolic events. However, there is a lack of consensus regarding the optimal type, dose, and especially duration of pharmacologic prophylaxis after bariatric surgery. An extended pharmacoprophylaxis has been suggested by various authors.^14,15,16^ Depending on the estimated VTE risk and country-specific recommendations, recommendations for VTE prophylaxis after bariatric surgery range from 7 days to 4 weeks.^17,18,19^ Whether extended prophylaxis decreases the risk of VTE events in clinical practice has not been well investigated in prospective randomized studies.

With the benefits of oral dosing, low drug-drug interaction potential, no known food interaction, and fixed dosing regimens, direct oral anticoagulants (DOACs) have expanded the spectrum of anticoagulants during the last several years. Once-daily oral rivaroxaban is approved for primary (and secondary) thromboprophylaxis in patients with elective hip or knee replacement surgery,^20,21,22^ without the need for dose adjustment in patients with extreme obesity.^23^ Clinical data on the use of DOACs in patients with obesity are scarce. An in vitro study in this patient population documented a rivaroxaban concentration–dependent inhibition of in vivo thrombin generation.^24^ Nevertheless, there are currently no guidelines available to guide DOAC use in patients undergoing bariatric surgery.

The impact of bariatric surgery with a consecutively altered anatomy on pharmacokinetics and pharmacodynamics of a single prophylactic dose of rivaroxaban has been investigated in 2 previous phase 1 clinical trials.^25,26^ In these studies, altered postbariatric anatomy and weight loss did not affect pharmacokinetic and pharmacodynamic parameters of rivaroxaban in a clinically relevant way. However, to our knowledge, no prospective randomized clinical trial has investigated the use of DOACs after upper gastrointestinal tract surgery, including bariatric procedures. Therefore, we designed and conducted the BARIVA (Bariatric Rivaroxaban) trial to investigate the efficacy and safety of a prophylactic dose of rivaroxaban in a randomized setting (7- vs 28-day prophylaxis) in patients after bariatric surgery.

## Methods

### Study Design

The BARIVA study was designed as an investigator-initiated, assessor-blinded, multicenter, phase 2 randomized clinical trial with a calculated number of 130 patients in each of the 2 study groups: short vs long prophylaxis with rivaroxaban in patients with severe obesity after bariatric surgery. The study was conducted at 3 bariatric centers in Switzerland from July 1, 2018, to June 30, 2021. All patients provided written informed consent. The trial was performed according to the Declaration of Helsinki,^27^ the guideline for Good Clinical Practice E6, and applicable local laws and regulations. The study protocol was approved by the independent ethics committee of the Canton Bern before the initiation of the study, and the study was registered in the ClinicalTrials.gov registry (NCT03522259). The protocol is provided in Supplement 1. This study followed the Consolidated Standards of Reporting Trials (CONSORT) guideline.

### Study Population

According to the criteria for bariatric surgery in Switzerland, study inclusion criteria were a body mass index (BMI; calculated as weight in kilograms divided by height in meters squared) greater than 35, an age of 18 years or older, and failure of conservative treatment for 2 years. Participant race and ethnicity were documented by clinicians to show the demographic balance of the study population. Patients were ineligible if they were pregnant or breastfeeding, had active bleeding or a high risk of bleeding, or had a history of VTE (for additional exclusion criteria, see eMethods 1 in Supplement 2). Assignment of patients to the different study populations of the trial is shown in the Figure.

**Figure.**
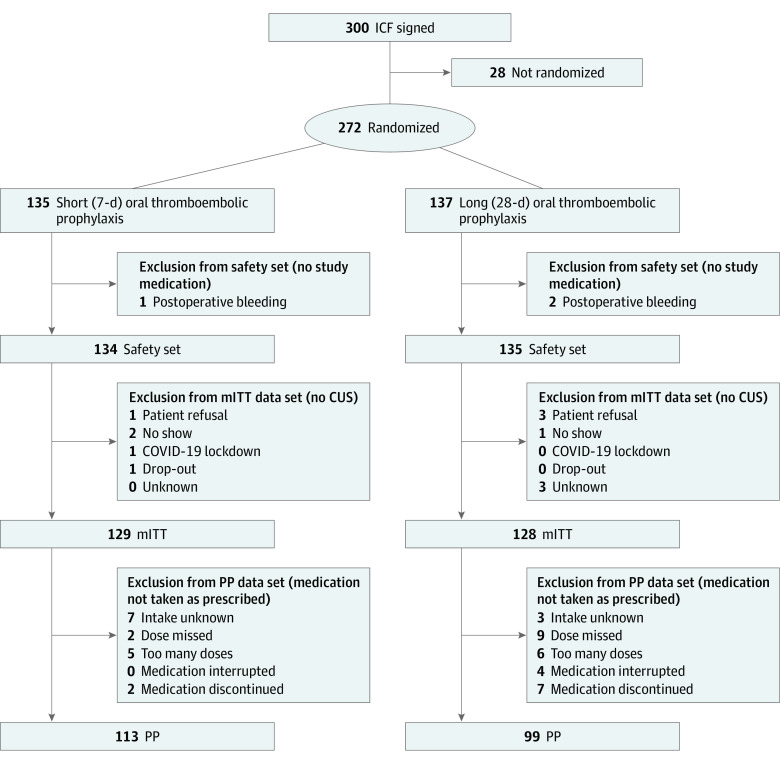
Trial Profile CUS indicates compression ultrasonography screening; ICF, informed consent form; mITT, modified intention to treat; and PP, per protocol.

### Study Procedures

Selection of the bariatric procedures was independent of this study. Standard postoperative thromboprophylaxis and nutrition management are described in eMethods 2 in Supplement 2. The randomization to 1 of the 2 rivaroxaban groups was performed on postoperative day 1 after exclusion of a clinically relevant bleeding or a suspected stenosis. Patients were randomly assigned 1:1 either to 7 days (short prophylaxis) or 28 days (long prophylaxis) of oral thromboembolic prophylaxis. Randomization was performed via an electronic online randomization system using different block sizes. Patients were stratified according to the surgical procedure (Roux-en-Y gastric bypass, sleeve gastrectomy [SG], or revisional surgery), sex, and study center.

Enrolled patients received an oral dose of 10 mg of rivaroxaban (Xarelto, Bayer Pharma AG) under nonfasting conditions on the first postoperative day. Rivaroxaban, 10 mg/d, was selected based on our 2 previous phase 1 pharmacokinetic/pharmacodynamic trials and the in vitro thrombin generation study.^24,25,26^ Rivaroxaban was continued for 7 days in the short prophylaxis group and for 28 days in the long prophylaxis group. All patients had a bilateral compression ultrasonography screening (CUS) of both legs after 28 days (with a window of ±2 days) by a vascular ultrasonography specialist who was blinded regarding the allocated treatment group and a clinical follow-up visit. A safety follow-up visit at day 35 (1 week after the CUS assessment) was performed by telephone.

Pulmonary embolism was assessed based on clinical signs and symptoms during hospitalization and during the follow-up visits. Suspected PE was confirmed by contrast-enhanced spiral computed tomography or ventilation/perfusion scintigraphy.

### Study Outcomes

The primary outcome was a composite of any DVT (proximal and distal as well as asymptomatic and symptomatic) or objectively confirmed PE. Secondary outcomes were the incidence of DVT (asymptomatic and symptomatic) during treatment and follow-up and all-cause mortality during the follow-up period (within 28 ± 2 days after surgery). Postoperative morbidity was also recorded by using the Clavien-Dindo classification.^28^

The main safety outcome was the incidence of major bleeding defined by the International Society on Thrombosis and Hemostasis (ISTH; bleeding leading to transfusion or a decrease in the hemoglobin level of ≥2 g/dL [to convert to grams per liter, multiply by 10]) during the intervention and observation periods. Secondary safety end points were the percentages of patients with ISTH-defined clinically relevant nonmajor bleeding, cardiovascular events, drug allergy and drug sensitivity, and death.^29^

### Statistical Analysis

The aim of this study was to estimate the proportion of 28-day VTE in both treatment groups with a given precision. No formal hypothesis testing between the 2 treatment groups was intended. The sample size was assessed based on simulations (eMethods 3 in Supplement 2). The baseline characteristics and surgery details were compared between treatment groups by an independent sample, unpaired, 2-tailed *t* test, a Mann-Whitney *U* test, or a χ^2^ test, as appropriate.

The safety population consisted of all patients who received at least 1 dose of study medication. The modified intention-to-treat (mITT) population consisted of all patients who were valid for the safety analysis and who had the full outcome assessment for the primary outcome measure of thromboembolism (for additional statistical methods, see eMethods 3 in Supplement 2). All statistical analyses were performed in R, version 4.1.2 (R Foundation for Statistical Computing).

## Results

### Study Population and Trial Profile

Of 269 patients in this clinical trial (mean [SD] age, 40.0 [12.1] years; 216 female [80.3%] and 53 male [19.7%]; mean body mass index, 42.2), 134 patients (50.0%) received a 7-day and 135 patients (50%) received a 28-day VTE prophylaxis course with rivaroxaban. Baseline and surgical characteristics are presented in Table 1. The 2 treatment groups were well balanced, with the exception of diabetes and smoking, which were both more frequently observed in the short prophylaxis group.

**Table 1.** Baseline and Surgical Characteristics of Patients^a^

Characteristic	Overall (N = 269)	Rivaroxaban 7 d (n = 134)	Rivaroxaban 28 d (n = 135)	*P* value
Sex				
Female	216 (80.3)	108 (80.6)	108 (80.0)	>.99
Male	53 (19.7)	26 (19.4)	27 (20.0)	
Age, mean (SD), y	40.0 (12.1)	39.9 (12.1)	40.1 (12.2)	.94
Height, mean (SD), m	1.7 (0.1)	1.7 (0.1)	1.7 (0.1)	.91
Weight, mean (SD), kg	117.8 (21.0)	116.5 (19.4)	119.1 (22.5)	.32
BMI, mean (SD)	42.2 (5.9)	41.7 (5.1)	42.6 (6.5)	.21
Race and ethnicity				
Asian	5 (1.9)	2 (1.5)	3 (2.2)	.55
Black	4 (1.5)	3 (2.2)	1 (0.7)	
White	260 (96.7)	129 (96.3)	131 (97.0)	
Heart disease				
No	254 (94.4)	128 (95.5)	126 (93.3)	.61
Yes	15 (5.6)	6 (4.5)	9 (6.7)	
Diabetes				
No	236 (87.7)	111 (82.8)	125 (92.6)	.02
Yes	33 (12.3)	23 (17.2)	10 (7.4)	
OSAS				
No	189 (70.3)	91 (67.9)	98 (72.6)	.48
Yes	80 (29.7)	43 (32.1)	37 (27.4)	
GERD				
No	190 (70.6)	95 (70.9)	95 (70.4)	>.99
Yes	79 (29.4)	39 (29.1)	40 (29.6)	
Smoker				
No	190 (70.6)	85 (63.4)	105 (77.8)	.01
Yes	79 (29.4)	49 (36.6)	30 (22.2)	
ASA score				
1	1 (0.4)	1 (0.7)	0	.45
2	68 (25.3)	31 (23.1)	37 (27.4)	
3	200 (74.3)	102 (76.1)	98 (72.6)	
Type of bariatric surgery				
RS	17 (6.3)	10 (7.5)	7 (5.2)	.72
RYGB	138 (51.3)	69 (51.5)	69 (51.1)	
SG	114 (42.4)	55 (41.0)	59 (43.7)	
Duration of surgery, median (IQR), min	67 (45-100)	69 (45-110)	65 (45-100)	.65
Duration of hospitalization, median (IQR), d	3 (3-4)	3 (3-4)	3 (3-4)	.84

Abbreviations: ASA, American Society of Anesthesiology Physical Status Classification System; BMI, body mass index (calculated as weight in kilograms divided by height in meters squared); GERD, gastroesophageal reflux disease; OSAS, obstructive sleep apnea syndrome; RS, revisional surgery; RYGB, Roux-en-Y gastric bypass; SG, sleeve gastrectomy.

^a^ Data are presented as number (percentage) of patients unless otherwise indicated.

The mITT population consisted of 257 patients. Of these, 129 patients (50.0%) were in the short prophylaxis group and 128 patients (50.0%) were in the long prophylaxis group. Because of a missing CUS, 12 patients had to be excluded from the mITT population.

A total of 300 patients consented to study participation. Twenty-eight (9.3%) were excluded before randomization for several reasons, including cancellation of surgery, screening failure, patient withdrawal, or withdrawal because of perioperative complications that precluded the timely start of study medication. In total, 272 patients (90.7%) were randomized. Of these, 3 patients (1.1%) were excluded from the safety analysis because of immediate postoperative bleeding complications (these patients had no exposition to rivaroxaban).

### Efficacy Outcomes

In 257 patients in the mITT data set, only 1 primary efficacy end point was observed (0.4%; 95% CI, 0.02%-2.2%). This single primary end point event concerned an asymptomatic DVT in a patient undergoing SG in the long prophylaxis group (eFigure 1 in Supplement 2). No patient had a clinically overt DVT or PE.

### Safety Outcomes and Secondary Outcomes

Major or clinically relevant nonmajor bleeding events were observed in 5 patients (1.9%): 2 in the short prophylaxis group and 3 in the long prophylaxis group. The difference between the groups was not statistically significant. Details are specified in Table 2 and eFigure 2 in Supplement 2. Clinically nonsignificant bleeding events were observed in 10 patients (3.7%): 3 in the short prophylaxis group and 7 in the long prophylaxis group. Details are listed in eTable 1 in Supplement 2.

**Table 2.** Secondary Outcomes: Major Bleeding and Clinically Relevant Nonmajor Bleeding

End point	No. (%; 95% CI)^a^		
	Overall	Rivaroxaban 7 d	Rivaroxaban 28 d
**Total population**			
Total No.	269	134	135
Primary safety end point: major bleeding	2 (0.7; 0.2-2.7)	1 (0.7; 0.0-4.1)	1 (0.7; 0.0-4.1)
Secondary safety end point: clinically relevant nonmajor bleeding	3 (1.1; 0.4-3.2)	1 (0.7; 0.0-4.1)	2 (1.5; 0.4-5.2)
Major or clinically relevant nonmajor bleeding	5 (1.9; 0.8-4.3)	2 (1.5; 0.4-5.3)	3 (2.2; 0.8-6.3)
**RYGB**			
Total No.	138	69	69
Primary safety end point: major bleeding	1 (0.7; 0.0-4.0)	0 (0; 0-5.3)	1 (1.4; 0.1-7.8)
Secondary safety end point: clinically relevant nonmajor bleeding	3 (2.2; 0.7-6.2)	1 (1.4; 0.1-7.8)	2 (2.9; 0.8-10.0)
Major or clinically relevant nonmajor bleeding	4 (2.9; 1.1-7.2)	1 (1.4; 0.1-7.8)	3 (4.3; 1.5-12.0)
**SG**			
Total No.	114	55	59
Primary safety end point: major bleeding	1 (0.9; 0.0-4.8)	1 (1.8; 0.1-9.6)	0 (0; 0-6.1)
Secondary safety end point: clinically relevant nonmajor bleeding	0 (0; 0-3.3)	0 (0; 0-6.5)	0 (0; 0-6.1)
Major or clinically relevant nonmajor bleeding	1 (0.9; 0.1-4.8)	1 (1.8; 0.1-9.6)	0 (0; 0-6.1)
**RS**			
Total No.	17	10	7
Primary safety end point: major bleeding	0 (0; 0-18.4)	0 (0; 0-27.8)	0 (0; 0-35.4)
Secondary safety end point: clinically relevant nonmajor bleeding	0 (0; 0-18.4)	0 (0; 0-27.8)	0 (0; 0-35.4)
Major or clinically relevant nonmajor bleeding	0 (0; 0-18.4)	0 (0; 0-27.8)	0 (0; 0-35.4)

Abbreviations: RS, revisional surgery; RYGB, Roux-en-Y gastric bypass; SG, sleeve gastrectomy.

^a^ To calculate the 95% CIs where the number events was 0, a continuity correction was applied (eMethods 3 in Supplement 2).

### Postoperative Complications

Postoperative complications (except bleeding events) are listed in Table 3. In the short prophylaxis group, 7 events were observed; in the long prophylaxis group, 17 events were observed. However, this difference was not statistically significant. No cardiovascular or cerebral ischemia events and no fatal outcomes were reported during the study. No study medication–associated grade 4 or 5 adverse events occurred (Table 3; eTable 2 in Supplement 2). Postsurgery complications were classified according to the Clavien-Dindo classification^28^ and are listed in Table 3.

**Table 3.** Postoperative Complications

Postoperative complication	No. (%; 95% CI)^a^		
	Overall (N = 257)	Rivaroxaban 7 d (n = 129)	Rivaroxaban 28 d (n = 128)
Asymptomatic VTE	1 (0.4; 0.02-2.2)	0 (0.0; 0.0-2.9)	1 (0.8; 0.0-4.3)
Postoperative complications			
Any postoperative complication	24 (9.3; 6.4-13.5)	7 (5.4; 2.7-10.8)	17 (13.3; 8.5-20.2)
Superficial SSI	2 (0.8; 0.2-2.8)	1 (0.8; 0.0-4.3)	1 (0.8; 0.0-4.3)
Deep SSI	1 (0.4; 0.0-2.2)	0 (0.0; 0.0-2.9)	1 (0.8; 0.0-4.3)
Organ-space SSI	5 (1.9; 0.8-4.5)	2 (1.6; 0.4-5.5)	3 (2.3; 0.8-6.7)
Wound dehiscence	3 (1.2; 0.4-3.4)	2 (1.6; 0.4-5.5)	1 (0.8; 0.0-4.3)
Deep venous thrombosis	1 (0.4; 0.0-2.2)	0 (0.0; 0.0-2.9)	1 (0.8; 0.0-4.3)
Urinary tract infection	2 (0.8; 0.2-2.8)	0 (0.0; 0.0-2.9)	2 (1.6; 0.4-5.5)
Readmission	4 (1.6; 0.6-3.9)	0 (0.0; 0.0-2.9)	4 (3.1; 1.2-7.8)
Reoperation	1 (0.4; 0.0-2.2)	1 (0.8; 0.0-4.3)	0 (0.0; 0.0-2.9)
Other	5 (1.9; 0.8-4.5)	1 (0.7; 0.0-4.3)	4 (3.1; 1.2-7.8)
Clavien-Dindo classification			
Grade I	9 (3.5; 1.9-6.5)	4 (3.1; 1.2-7.7)	5 (3.9; 1.7-8.8)
Grade II	10 (3.9; 2.1-7.0)	2 (1.6; 0.4-5.5)	8 (6.3; 3.2-11.8)
Grade IIIa	4 (1.6; 0.6-3.9)	0 (0.0; 0.0-2.9)	4 (3.1; 1.2-7.8)
Grade IIIb	1 (0.4; 0.0-2.2)	1 (0.8; 0.0-4.3)	0 (0.0; 0.0-2.9)
Allergic reactions	6 (2.3; 1.1-5.0)	3 (2.3; 0.8-6.6)	3 (2.3; 0.8-6.7)

Abbreviations: SSI, surgical site infection; VTE, venous thromboembolism.

^a^ To calculate the 95% CIs where the number events was 0, a continuity correction was applied (eMethods 3 in Supplement 2).

### Adverse Events

In total, 72 adverse events occurred in 58 patients (21.6%). Of these, 19 were serious adverse events, and 12 patients discontinued study medication because of an adverse event (eTable 2 in Supplement 2). The rate of suspected allergic reaction was similar in both treatment groups (Table 3). Bleeding events and postoperative complications were further analyzed by subgroups (ie, SG, Roux-en-Y gastric bypass, and revisional surgery) (eTable 3 in Supplement 2).

## Discussion

To our knowledge, this is the first trial in which an oral factor Xa inhibitor has been prospectively investigated in patients after gastrointestinal surgery in general and in bariatric surgery in particular. The results of this randomized clinical trial indicate that thromboprophylaxis with 10 mg/d of rivaroxaban is efficacious and safe in the postoperative setting of bariatric surgery. The overall number of primary end point events was very low (1 single asymptomatic DVT in a patient undergoing SG with 28-day prophylactic treatment). Therefore, no significant difference between the 2 treatment groups regarding the primary composite end point (symptomatic or asymptomatic DVT and PE) could be detected. Although the altered anatomy after bariatric surgery is of some theoretical concern, no relevant effect on pharmacokinetic parameters could be observed in previous phase 1 studies.^25,30^

The subgroup analysis did not reveal any VTE difference among patients undergoing SG, Roux-en Y gastric bypass, and revisional surgery or within the same subgroup based on duration of prophylaxis (eTable 2 and eDiscussion 1 in Supplement 2). Consequently, type of surgical procedure does not seem to influence outcome of thromboembolic prophylaxis regarding efficacy. No patient presented with clinically overt signs or symptoms characteristic of PE.

Of note, this study was not designed to prove equivalence between the 2 treatment durations, and no equivalence testing has been planned or performed. Testing for equivalence would have required a larger study population.

Given the very low number of thromboembolic events in both treatment groups, assessment of bleeding events was of special interest. The number of major bleeding events and clinically relevant nonmajor bleedings was low in the 7- and 28-day treatment groups (2 and 3 events, respectively), a difference that was not statistically significant. Although postoperative complications (including bleeding events) were more frequent in the long prophylaxis group, this difference was also not statistically significant.

Low-molecular-weight heparins (LMWHs) are the most frequently prescribed pharmacologic prophylaxis for VTE in patients undergoing bariatric surgery.^31^ However, LMWHs as VTE prophylaxis were investigated only in a few randomized clinical trials in these patients. Comparing our results to LMWHs is difficult because some of these trials had a biochemical or a pharmacological primary end point rather than a clinical end point and the number of patients enrolled was usually small. Moreover, type of LMWH, dosage, and initiation and duration of pharmacoprophylaxis differed from trial to trial.^32,33,34^

Our observations are consistent with those of 3 randomized clinical trials^35,36,37^ evaluating the efficacy and safety of LMWHs in patients undergoing bariatric surgery. In these trials, VTE occurred in 0% to 1.5% of patients, and the corresponding rates of bleeding complications were 5% to 6.7%.^35,36,37^ Although most postdischarge VTE events occur within 30 days after the bariatric intervention,^7,11^ the effectiveness of extended prophylactic anticoagulation up to 30 days has not been extensively investigated in prospective clinical trials,^9,38^ and the optimal duration of anticoagulation is unknown, resulting in differing recommendations in guidelines.

In a prospective trial of 308 patients undergoing bariatric surgery, Raftopoulos et al^14^ studied the effect of continued prophylaxis after hospital discharge (10 additional days of LMWH) vs in-hospital thromboprophylaxis only. The rate of VTE in the long prophylaxis group was significantly lower compared with the group who received only in-hospital prophylaxis (0% vs 4.5%; *P* = .006). Depending on the VTE risk, some bariatric centers propose thromboprophylaxis for 1 week up to 4 weeks after bariatric surgery.^9,17^

Our clinical trial results did not show a significant difference in thromboembolic events between the 2 treatment groups. Therefore, prophylaxis for 7 days after bariatric surgery might be sufficient to prevent thromboembolic events in most patients with a moderate risk of VTE. According to the recently published American Society for Metabolic and Bariatric Surgery recommendations on perioperative thromboprophylaxis in patients undergoing bariatric surgery, almost all patients are considered to be at least at moderate risk for VTE events.^13^ Depending on the constellation of patient- and procedure-related risk factors, some patients undergoing bariatric surgery are at higher or very high risk for the development of postoperative VTE.^9^ In the current study, patients at higher risk for VTE (eg, with a history of VTE) were excluded. Importantly, even with prophylaxis for 28 days, thromboembolic events could not be prevented entirely. Some authors used risk-adjusted approaches to VTE prophylaxis, but these algorithms are not well established, and randomized clinical trials to determine the optimal duration of VTE prophylaxis after bariatric surgery are missing^9,39,40,41^ (eDiscussion 2 in Supplement 2).

Data regarding prolonged use of prophylactic DOACs after surgical interventions are limited, except for data on major orthopedic surgery.^42^ A previous study^43^ compared the effect of prolonged VTE prophylaxis with either apixaban or enoxaparin for up to 28 days in a gynecologic oncology population. The rates of VTE did not differ between the groups (1.0% in the apixaban vs 1.5% in the enoxaparin group, *P* = .68), and the rates of major bleeding events (0.5% vs 0.5%, *P* > .99) as well as clinically relevant nonmajor bleeding events (5.4% vs 9.7%, *P* = .11) were not statistically different.

Two previous phase 1 clinical trials^25,26^ demonstrated that pharmacokinetic and pharmacodynamic parameters of rivaroxaban are not significantly altered in patients with severe obesity in the early phase and several months after bariatric surgery. In the current study, 164 patients (61.0%) of the safety population had a body weight greater than 120 kg, and 148 (55.0%) had a BMI greater than 40. In contrast to the recommendations of the ISTH^44,45,46,47^ (eDiscussion 3 in Supplement 2), results of this trial confirm that thromboembolic prophylaxis with the DOAC rivaroxaban for 7 days and for 28 days is efficacious and safe in this study population, including patients with a body weight greater than 120 kg and/or a BMI greater than 40. Overall, thromboembolic prophylaxis with DOACs is less expensive than with LMWHs and more convenient for patients because no subcutaneous injections are required.

### Strengths and Limitations

A strength of this trial was detecting asymptomatic thrombotic events by CUS. This is important because 10% of untreated thrombotic events can result in symptomatic pulmonary embolism.^48,49^ However, systematic thrombosis screening with CUS outside the context of clinical trials is usually not performed. Thromboembolic events that occurred during the trial period were treated according to local clinical practice. Whether DOACs are suitable to treat thromboembolic events in patients undergoing bariatric surgery cannot be answered with this trial and should be further investigated in a prospective clinical trial.

This study has some limitations. The main limitation is the lack of an LMWH treatment arm. Therefore, a direct comparison of thromboembolic prophylaxis between rivaroxaban and LMWH is not possible. However, the results of this trial are comparable to those of trials investigating LMWH as thromboembolic prophylaxis regarding efficacy and safety. The very low number of primary outcome events was unexpected, especially because asymptomatic DVT was part of the composite end point. Because of the single primary composite outcome event, a direct comparison of the 2 treatment groups regarding efficacy is difficult. In addition, the number of patients investigated in this trial was relatively small, compared with a larger phase 3 trial. However, this is the first randomized clinical trial investigating thromboembolic prophylaxis with a DOAC in the context of bariatric surgery, and the inclusion of CUS to detect asymptomatic DVT increases the validity of the efficacy results.

## Conclusions

In this phase 2 randomized clinical trial, postbariatric thrombosis prophylaxis with 10 mg/d of rivaroxaban was associated with a very low incidence of thromboembolic events irrespective of treatment duration. The number of safety events was higher in the long prophylaxis group compared with the short prophylaxis group, but this difference was not statistically significant. Our data suggest that thromboembolic prophylaxis with 10 mg of rivaroxaban for 7 days after bariatric surgery is efficacious and safe. A larger prospective trial in patients undergoing bariatric surgery comparing thromboembolic prophylaxis with short-term DOAC and extended LMWH treatment would be valuable to confirm the results of this study and to guide future prophylactic treatment.

## Notes

Supplement 1.
Trial Protocol

Supplement 2. **eMethods 1.** In- and Exclusion Criteria **eMethods 2.** Standard Postoperative Thromboprophylaxis and Nutrition Management **eMethods 3.** Statistical Analysis **eDiscussion 1.** Subgroups **eDiscussion 2.** Risk-Adjusted Approaches for Duration of Thromboembolic Prophylaxis **eDiscussion 3.** Statement of the ISTH **eTable 1.** Clinically Non-relevant Bleedings **eTable 2.** Postoperative Complications & Adverse Events **eTable 3.** Overview of Secondary Outcome in Patients Who Underwent RYGB, SG and RS (Safety Population) **eFigure 1.** Cumulative Event Rate of Symptomatic and Asymptomatic VTE (iMTT Data Set, n=257) **eFigure 2.** Cumulative Event Rate of Any Major and Clinically Relevant Non-Major Bleeding (Safety Population, n=269)

Supplement 3.
Data Sharing Statement
