# Supplementary material for: Efficacy and Safety of Rivaroxaban for Postoperative Thromboprophylaxis in Patients After Bariatric Surgery: A Randomized Clinical Trial
Source: JAMA Netw Open. 2023 May 25;6(5):e2315241. doi: 10.1001/jamanetworkopen.2023.15241 (PMC10214035; doi:10.1001/jamanetworkopen.2023.15241)
Supplement: Supplement 1. — Trial Protocol [file jamanetwopen-e2315241-s001.pdf]

Clinical Study Protocol

BARIVA: Short versus extended prophylaxis of rivaroxaban for venous thromboembolism after bariatric surgery - a randomized controlled multicentre trial

Short title: Rivaroxaban as thrombosis prophylaxis in bariatric surgery

Study Type:	Clinical trial with Investigational Medicinal Product (IMP) Study
Categorisation:	Risk category according to LHR: B
Study Registration:	SNCTP ClinicalTrials.gov
Study Identifier:	UVCMBS003
Sponsor:	Inselspital Bern, CH-3010 Bern
Represented by:	Dr. med. Dino Kröll, UVCM, Inselspital, CH-3010 Bern
Principal Investigator:	Dr. med. Dino Kröll, UVCM, Inselspital, CH-3010 Bern
Investigational Product:	Rivaroxaban (Xarelto®)
Protocol Version and Date:	Version 1.3 of February 15, 2018

CONFIDENTIAL

The information contained in this document is confidential and the property of the sponsor. The information may not - in full or in part - be transmitted, reproduced, published, or disclosed to others than the applicable Competent Ethics Committee(s) and Regulatory Authority(ies) without prior written authorisation from the sponsor except to the extent necessary to obtain informed consent from those who will participate in the study. Exeption authorized by Dr. Kröll: relevant protocol reviewers at Bayer Pharma AG and Janssen Pharmaceuticals, Inc.

Signature Page

Study number **UVCMBS003**

Study Title **BARIVA: Short versus extended prophylaxis of rivaroxaban for venous thromboembolism after bariatric surgery - a randomized controlled multicentre trial**

Sponsor-Investigator and study team have approved the protocol version 1.3 (dated 15.02.2018), and confirm hereby to conduct the study according to the protocol, current version of the World Medical Association Declaration of Helsinki, ICH-GCP guidelines and the local legally applicable requirements [1-5].

Sponsor-Investigator & Principal Investigator Study Site Inselspital, Bern:

Dr. med. Dino Kröll

______________________ ______________________

Place/Date Signature

Clinical Pharmacologist:

Dr. med. Guido Stirnimann

______________________ ______________________

Place/Date Signature

Principal Investigator Study Site Beau Site, Bern:

Dr. med. Joerg Zehetner, Professor (USC)

______________________ ______________________

Place/Date Signature

Principal Investigator Study Site Kantonsspital Baden, Baden:

Prof. Dr. med. A. Nocito

______________________ ______________________

Place/Date Signature

Table of Contents

Signature Page 2
Table of Contents 3
STUDY SYNOPSIS 6
STUDY SUMMARY IN LOCAL LANGUAGE 10
ABBREVIATIONS 10
STUDY SCHEDULE 11
1. STUDY ADMINISTRATIVE STRUCTURE 12
1.1 Sponsor 12
1.2 Coordinating Investigator 12
1.3 Local Principal Investigators 12
1.4 Clinical Pharmacologist 12
1.5 Hemostasis Specialist 13
1.6 Statistician 13
1.7 Laboratories 13
1.8 Monitoring Institution 13
1.9 Data Safety Monitoring Committee 14
2. ETHICAL AND REGULATORY ASPECTS 15
2.1 Study registration 15
2.2 Categorisation of study 15
2.3 Competent Ethics Committee (CEC) 15
2.4 Competent Authorities (CA) 15
2.5 Ethical Conduct of the Study 15
2.6 Declaration of interest 15
2.7 Patient Information and Informed Consent 15
2.8 Participant privacy and confidentiality 15
2.9 Early termination of the study 16
2.10 Protocol amendments 16
3. BACKGROUND AND RATIONALE 16
3.1 Background and Rationale 16
3.2 Investigational Product (treatment) and Indication 17
3.3 Preclinical Evidence 17
3.4 Clinical Evidence to Date 17
3.5 Dose Rationale 18
3.6 Explanation for choice of comparator (or placebo) 18
3.7 Risks / Benefits 18
3.8 Justification of choice of study population 18
4. STUDY OBJECTIVES 19
4.1 Overall Objective 19
4.2 Primary Objective 19
4.3 Secondary Objectives 19
4.4 Safety Objectives 19
5. STUDY OUTCOMES 19

5.1 Primary Outcome 19
5.2 Secondary Outcomes 19
5.3 Other Outcomes of Interest 20
5.4 Safety Outcomes 20
6. STUDY DESIGN 20
6.1 General study design and justification of design 20
6.2 Methods of minimising bias 21
6.3 Unblinding Procedures (Code break) 21
7. STUDY POPULATION 21
7.1 Eligibility criteria 21
7.2 Recruitment and screening 22
7.3 Assignment to study groups 22
7.4 Criteria for withdrawal / discontinuation of participants 23
8. STUDY INTERVENTION 23
8.1 Identity of Investigational Product (treatment) 23
8.2 Administration of experimental and control interventions 23
8.3 Dose 23
8.4 Compliance with study intervention 24
8.5 Data Collection and Follow-up for withdrawn participants 24
8.6 Trial specific preventive measures 24
8.7 Concomitant interventions (treatments) 24
8.8 Study Drug Accountability 24
8.9 Return or Destruction of Study Drug 24
9. STUDY ASSESSMENTS 24
9.1 Study flow chart 24
9.2 Table of study procedures and assessments 25
9.3 Assessments of outcomes 25
9.4 Procedures at each visit 27
10. SAFETY 30
10.1 Collection and assessment of safety information 30
11. STATISTICAL METHODS 32
11.1 Hypothesis 32
11.2 Determination of Sample Size 32
11.3 Statistical criteria of termination of trial 33
11.4 Planned Analyses 33
11.5 Handling of missing data and drop-outs 34
12. QUALITY ASSURANCE AND CONTROL 34
12.1 Data handling and record keeping / archiving 34
12.2 Data management 35
12.3 Monitoring 35
12.4 Audits and Inspections 35
12.5 Confidentiality, Data Protection 35
12.6 Storage of biological material and related health data 35
13. PUBLICATION AND DISSEMINATION POLICY 35
14. FUNDING AND SUPPORT 36

14.1 Funding 36
14.2 Other Support 36
15. INSURANCE 36
16. REFERENCES 37
17. APPENDICES 39
17.1 Summary of product characteristics 39
17.2 Processing of study blood samples 41
17.3 Packaging, Labelling and Supply (re-supply) 42
17.4 Standardized Duplex Ultrasound Reporting 43

STUDY SYNOPSIS

**Sponsor:**	Sponsor: Inselspital, 3010 Bern<br>Responsible person: Dr. med. Dino Kröll
**Study Title:**	BARIVA: Short versus extended prophylaxis of rivaroxaban for venous thromboembolism after bariatric surgery - a randomized controlled trial
**Short Title / Study ID:**	Rivaroxaban as thrombosis prophylaxis in bariatric patients
**Protocol Version and Date:**	Version 1.2 of October 20, 2017
**Trial registration:**	SNCTP (Swiss National Clinical Trial Portal) ClinicalTrials.gov
**Study category and Rationale**	According to the Swiss legislation, this is a risk category B clinical trial: rivaroxaban is approved for thrombosis prophylaxis in orthopedic surgery. This trial investigates a new indication, i.e. thrombosis prophylaxis in bariatric surgery.
**Clinical Phase:**	This is a phase 2 clinical trial addressing venous thromboembolism and pharmacologic and pharmacodynamics parameters.
**Background and Rationale:**	Obesity is a risk factor for the development of venous thromboembolism and the association between obesity and postoperative VTE is well established. Given the significant VTE risk in bariatric patients, both the American College of Chest Physicians and the American Society for Metabolic and Bariatric Surgery have recommended, that in addition to mechanical prophylaxis some form of pharmacoprophylaxis should be administered to all bariatric surgery patients.<br>However, the pharmacoprophylaxis is not specified in detail in these recommendations. The aim of this study is to investigate the efficacy and safety of rivaroxaban in obese patients undergoing bariatric surgery. Rivaroxaban as an oral anticoagulant could be an attractive option for thromboprophylaxis compared to subcutaneous standard treatment after bariatric surgery. Especially in high-risk patients where an extended duration of thromboprophylaxis after hospital discharge is recommended, an oral therapy would be desirable.
**Objective(s):**	**Primary objectives**<br>To assess the safety and feasibility of VTE prophylaxis with 10 mg rivaroxaban QD for 7 and 28 days, respectively<br>**Secondary objectives**<br>- To compare VTE prophylaxis with 10 mg rivaroxaban QD for 7 and 28 days, respectively, regarding thromboembolic events.<br>- To assess the postoperative rates of asymptomatic DVT, symptomatic DVT, symptomatic PE, bleeding events, and investigator-reported adverse events (morbidity and mortality).<br>- To assess the pharmacokinetic effect of 10 mg rivaroxaban after repeated administration in bariatric surgery (subgroup of patients)<br>- To assess the pharmacodynamic effect of 10 mg rivaroxaban after bariatric surgery (subgroup of patients)

**Outcome(s):**	**Primary outcomes**<br>Symptomatic or asymptomatic venous thromboembolism (VTE). Composite endpoint consisting of one of the following elements<br>- any deep vein thrombosis (proximal and distal); suspected symptomatic deep- vein thrombosis will be assessed by ultrasound<br>- objectively confirmed pulmonary embolism (PE); suspected pulmonary embolism will be confirmed by contrast-enhanced spiral CT<br>**Secondary outcomes**<br><u>Clinical outcome variables</u><br>- Symptomatic VTE within 28 days after bariatric surgery<br>- Asymptomatic VTE within 28 days after bariatric surgery<br>- All-cause mortality within 28 days after bariatric surgery<br>- Postoperative morbidity (Dindo-Clavien classification) within 28 days after bariatric surgery<br>- VTE-related death within 28 days postoperatively<br><u>Pharmacokinetic parameters (subgroup of 16 RYGB plus 16 SG rivaroxaban patients.</u><br>- Rivaroxaban area under plasma concentration curve (AUC)<br>- Maximum plasma concentration (C_{max}) of rivaroxaban<br>- Time of maximum plasma concentration (T_{max}) of rivaroxaban<br>- Rivaroxaban trough plasma concentration (C_{min}) (24 h post last application)<br><u>Pharmacodynamic parameters (subgroup of 16 RYGB plus 16 SG rivaroxaban patients.</u><br>- Prothrombin time (PT)<br>- Thrombin generation assay (TGA)<br>- Prothrombin fragment (F1+2)<br>- Thrombin-antithrombin-complexes (TAT)<br>- D-Dimers
**Study Design:**	Randomized, interventional, multicenter phase II safety and feasibility study with two treatment arms (short vs. extended prophylaxis with rivaroxaban)

**Inclusion/Exclusion Criteria:**	**Inclusion criteria**<br>- Patient with scheduled elective bariatric surgery or redo surgery after bariatric interventions:<br>- Roux-en-Y gastric bypass surgery (RYGB) or sleeve gastrectomy (SG) or<br>- conversion from SG to RYGB or<br>- conversion from gastric banding to SG or RYGB or<br>- revision of gastric pouch or gastrojejunal anastomosis or revision of jejuno-jejunostomy<br>- Patient aged 18 years or older<br>- BMI ≥ 35 kg/m2<br>- Women of child-bearing age: Willingness of using a double barrier contraception method during the study period<br>- Written, informed consent<br>**Exclusion criteria**<br>- DVT and/or PE in the patient history<br>- Myocardial infarction, transient ischemic attack or stroke within 6 months of study entry<br>- Active pathological bleeding<br>- Uncontrolled hypertension<br>- Severely impaired hepatic function (cirrhotic patients with Child B or C) or renal function (creatinine clearance <30 mL per min)<br>- Concomitant treatment with strong CYP3A4 inhibitors (e.g., ketoconazole, itraconazole, lopinavir, ritonavir, indinavir).<br>- Concomitant treatment with a P-glycoprotein inhibitor and weak or moderate CYP3A4 inhibitor (e.g., erythromycin, azithromycin, diltiazem, verapamil, quinidine, ranolazine, dronedarone, amiodarone, felodipine).<br>- Concomitant treatment with P-glycoprotein inducers and strong CYP3A4 inducers (e.g. carbamazepine, phenytoin, rifampin).<br>- Therapy with any other drugs that might affect the study outcome, including anticoagulants, aspirin or other antiplatelet agents, Factor Xa inhibitors other than the study medication, or any other drug affecting coagulation<br>- Positive pregnancy test, pregnancy or nursing women<br>- Congenital or acquired bleeding disorder<br>- Known intolerance of the study medication rivaroxaban
**Study Product, Measurements and Procedures:**	Study Product: Rivaroxaban<br>Measurements and Procedures: A single dose of prophylactic LMWH is given 6 h after closure of surgical site. After the bariatric surgery procedure, patients are allowed to drink. Patients change their diet from liquid to a soft/normal food within the next days after the bariatric procedure (independent of the type of procedure performed).<br>Rivaroxaban 10 mg per os is started on the first postoperative day (i.e. 24 h after the surgical intervention). Patients receive Rivaroxaban 10 mg QD for 7 days (short arm) or 28 days (long arm).<br>In a subgroup of study patients (patients from the University Hospital Inselspital, Bern) PK/PD parameters are assessed following the last intake of

	rivaroxaban at day 28.<br>All clinically thromboembolic events will be assessed by ultrasound or CT, respectively, as soon as apparent. In addition, patients are screened for VTE at day 28±2 by ultrasound to detect clinically inapparent thromboses.
**Control Intervention:**	This is an phase 2 trial without control intervention.
**Number of Participants with Rationale:**	This is a safety and feasibility study with the aim of estimating the proportion of 28-day VTE in both treatment arms with a given precision. There will be no formal hypothesis testing between the two treatment arms. All outcomes will be evaluated descriptively in each group presenting estimates with 95% confidence intervals.<br>We expect a proportion of 28-day VTE of 0.5% and 5% (each including asymptomatic VTE diagnosed with US) in the extended and short treatment arm, respectively. A sample of 130 patients in each group (260 in total) will result in a two-sided 95% Wilson confidence interval of 0.1-3.7% and 2.4-10.1%, respectively.<br>The PK/PD study will be performed with 16 (RYGB) plus 16 (SG) participants.
**Study Duration:**	Estimated duration from screening of the first patient to the finalized study report is 2 years.
**Study Schedule:**	Start screening: December 2017<br>FPFV: December 2017<br>LPLV: May 2019<br>Database cleaning and query management: June 2019<br>Statistical analysis: August 2019<br>Publication of results: December 2019
**Investigator(s):**	Dr. Dino Kröll, OA UVCM, Inselspital, CH-3010 Bern
**Study Centre(s):**	University Clinic for Visceral Surgery and Medicine, University Hospital Inselspital, 3010 Bern<br>Clinic Beau-Site, Visceral Therapy, 3010 Bern<br>Department of Surgery, Kantonsspital Baden AG, 5404 Baden
**Phase of Clinical Trial:**	This is a phase 2 clinical trial
**Statistical Considerations:**	The primary analysis is an intention-to-treat (ITT) analysis, i.e. all patients will be analysed in the group they were originally allocated to.<br>Secondary analyses will be performed per protocol (PP), i.e. only patients that in fact received the study treatment according to the randomization will be analysed.<br>Patient characteristics will be presented as frequency and percentage for binary variables, mean and standard deviation for continuous, normally distributed variables, and median and interquartile range for continuous, non-normally distributed variables.<br>The primary and all secondary binary outcomes will be evaluated descriptively in each group presenting proportions with 95% Wilson confidence intervals.<br>Additionally, we will compare the primary and all secondary binary outcomes using a Cochran-Mantel-Haenszel-test stratified for the stratification factors used in the randomization. These tests will be exploratory, not confirmatory.<br>Pharmacokinetic and pharmacodynamic parameters will be presented in each treatment group as median with interquartile range and mean with 95% confidence interval.
**GCP Statement:**	This study will be conducted in compliance with the protocol, the current version of the Declaration of Helsinki, the ICH-GCP as well as all national legal and regulatory requirements.

STUDY SUMMARY IN LOCAL LANGUAGE

Bei dieser klinischen Studie, die in mehreren Zentren für Übergewichtschirurgie durchgeführt wird, wird die Wirkung des Medikamentes Xarelto (Rivaroxaban) 10 mg zur Verhinderung (Prophylaxe) von Thrombosen und Lungenembolien im Anschluss an die Übergewichtschirurgie untersucht. Patientinnen und Patienten erhalten im Anschluss an die Operation entweder währen 7 oder während 28 Tagen das gerinnungshemmende Medikament Xarelto. Nach 28 Tagen erfolgt eine Kontrolle der Beinvenen mittels Ultraschall, um auch Thrombosen festzustellen, die nicht zu Beschwerden geführt haben. Bei einem Teil der Studienpatienten werden im Anschluss an die letzte Einnahme des Studienmedikaments über die Dauer von 24 Stunden zusätzliche Untersuchungen zur Konzentration und zur Wirkung von Xarelto durchgeführt.

ABBREVIATIONS

AE	Adverse Event
aPTT	Activated Partial Thromboplastin Time
ASA	American Society of Anesthesiologists physical status classification system
AUC	Area Under the Curve
BMI	Body Mass Index
CA	Competent Authority (e.g. Swissmedic)
CEC	Competent Ethics Committee
Cmax	Maximum Plasma Concentration
Cmin	Minimal Plasma Concentration
CRF	Case Report Form
ClinO	Ordinance on Clinical Trials in Human Research (in German: KlinV, in French: OClin)
CTCAE	Common terminology criteria for adverse events
DSUR	Development safety update report
DVT	Deep vein thrombosis
eCRF	Electronic Case Report Form
F1+2	Thrombin fragments
FAS	Full Analysis Set
GCP	Good Clinical Practice
GFR	Glomerular Filtration Rate (Creatinin clearance)
IB	Investigator's Brochure
HFG	Humanforschungsgesetz (Law on human research, LHR)
HMG	Heilmittelgesetz
HRA	Federal Act on Research involving Human Beings
IMP	Investigational Medicinal Product
IIT	Investigator-initiated Trial
ITT	Intention to treat
KlinV	Verordnung über klinische Versuche in der Humanforschung (in English: ClinO, in French OClin)
LMWE	Low Molecular Weight Heparin
PE	Pulmonary Embolism
PI	Principal Investigator

POD	Postoperative Day
PP	Per Protocol
PT	Prothrombin time
RYGB	Roux-en-Y gastric bypass
RYAS	Roux-en-Y gastric bypass Analysis Set
SDV	Source Data Verification
SG	Sleeve Gastrectomy
SGAS	Sleeve Gastrectomy Analysis Set
SOP	Standard Operating Procedure
SPC	Summary of product characteristics
SUSAR	Suspected Unexpected Serious Adverse Reaction
TAT	Thrombin-Antithrombin-Complexes
Tmax	Time of Maximum Plasma Concentration
TMF	Trial Master File
VTE	Venous Thromboembolism

STUDY SCHEDULE

Study period	Screening	Surgery		Postoperative																				
**Study visit**	**V0**			**V1**	**V2**	**V3**		**V4**		**V5**		**V6**		**V7**									**V8**	**V9**
**Study day**	-30 bis -1	0		1	2	3	4-7	8±2	9-14	15±2	16-20	21±2	22-27	28±2									V7+1	V7+7
**Hosp day**		1		2	3	4																		
**Time (24 h clock)**		8	18+	8	8	8	8	8		8		8		7	8	9	10	11	12	14	16	20	8	
**Relative time points for PK/PD (32 patients, 16 RYGB, 16 SG, Inselspital Bern only)****														T -1h	T 0	T 1h	T 2h	T 3h	T 4h	T 6h	T 8h	T 12h	T 24h	
Informed consent	x																							
Medical history	X														x									
Concomitant medication	x														x									
Demographics	x																							
Physical exam incl. weight and height	x														x									
Inclusion/Exclusion-criteria	x																							
Coagulation/hemostasis parameters (INR, Platelets)	x																							
Liver and kidney function	x																							
Beta-HCG	x																							
Bariatric Surgery		x++																						
Study inclusion				x																				
Randomization				x																				
Discharge (earliest point of time)					x																			
Study arms																								
A: Rivaroxaban short arm																								
LMWH in prophylactic dose (e.g. Clexane, Fraxiparine)			x																					
Rivaroxaban 10 mg p.o. QD				x	x	x	x																	
B: Rivaroxaban long arm																								
LMWH in prophylactic dose (e.g. Clexane, Fraxiparine)			x																					
Rivaroxaban 10 mg p.o. QD				x	x	x	x	x	x	x	x	x	x		x									
Duplex sonography																				x				
PK/PD substudy																								
Anti Xa activity**								x		x		x		x		x	x	x	x	x	x	x	x	
Rivaroxaban Concentration**								x		x		x		x		x	x	x	x	x	x	x	x	
Thrombin-Antithrombin-Complex (TAT)**														x		x	x	x	x	x	x	x	x	
Prothrombin-Fragments (F1 + F2)**														x		x	x	x	x	x	x	x	x	
Thrombin Generation**														x		x	x	x	x	x	x	x	x	
D-Dimers	x**			x**				x**		x**		x**		x		x**	x**	x**	x**	x**	x**	x**	x**	
AEs/SAEs			x	x	x	x		x		x		x		x	x	x	x	x	x	x	x	x	x	x*

+1st dose 6–8 h after wound closure or after adequate haemostasis has been achieved, subsequent doses at 8 am

++ Study independent bariatric surgery

* Telephone call

** Analysis PK/PD 32 patients

1. STUDY ADMINISTRATIVE STRUCTURE

1.1 Sponsor

University Hospital Inselspital, 3010 Bern, Switzerland

Responsible person: Dino Kröll, MD
University Clinic for Visceral Surgery and Medicine (UVCM)
University Hospital Inselspital, 3010 Bern, Switzerland
E-Mail: dino.kroell@insel.ch
Telephone: +41 31 632 24 03

1.2 Coordinating Investigator

Dino Kröll, MD
University Clinic for Visceral Surgery and Medicine (UVCM)
University Hospital Inselspital, 3010 Bern, Switzerland
E-Mail: dino.kroell@insel.ch
Telephone: +41 31 632 24 03

1.3 Local Principal Investigators

Dr. med. Dino Kröll
University Clinic for Visceral Surgery and Medicine (UVCM)
University Hospital Inselspital, 3010 Bern, Switzerland
E-Mail: dino.kroell@insel.ch
Telephone: +41 31 632 24 03

Dr. med. J. Zehetner, Professor (USC)
Visceral Therapy
Clinic Beau-Site Bern, 3013 Bern, Switzerland
E-Mail: joerg.zehetner@hirslanden.ch
Telephone +41 31 312 61 12

PD Dr. med. A. Nocito
Department of Surgery
Kantonsspital Baden AG, Im Ergel 1, 5404 Baden, Switzerland
E-Mail: antonio.nocito@ksb.ch
Telephone +41 56 486 30 02

1.4 Clinical Pharmacologist

Dr. med. Guido Stirnimann
University Clinic for Visceral Surgery and Medicine (UVCM)
University Hospital Inselspital, 3010 Bern, Switzerland
E-Mail: guido.stirnimann@insel.ch
Telephone: +41 31 632 47 13

1.5 Hemostasis Specialist

Prof. Dr. med. Lorenzo Alberio

Service et Laboratoire Central d'Hématologie

Centre hospitalier universitaire vaudois (CHUV), Rue du Bugnon 46

1011 Lausanne, Switzerland

E-Mail: Lorenzo.alberio@chuv.ch

Telephone.: +41 21 314 11 11

1.6 Statistician

Andreas Limacher

Head of Statistics and Methodology CTU Bern

Finkenhubelweg 11, CH 3012 Bern

E-Mail: andreas.limacher@ctu.unibe.ch

Telephone: +41 31 631 35 10

Sabine Schädelin, MSc

Statistician, Clinical Trial Unit

Departement Klinische Forschung

c/o Universitätsspital Basel

Spitalstrasse 12, CH 4031 Basel

E-Mail: sabine.schaedelin@usb.ch

Telephone +41 61 556 51 67

1.7 Laboratories

Universitätsinstitut für Klinische Chemie

University Hospital Inselspital

3010 Bern, Switzerland

Hämatologisches Zentrallabor

University Hospital Inselspital

3010 Bern, Switzerland

Service et Laboratoire Central d'Hématologie

Centre hospitalier universitaire vaudois (CHUV)

Rue du Bugnon 46

1011 Lausanne, Switzerland

1.8 Monitoring Institution

CTU Bern

Finkenhubelweg 11

3012 Bern, Switzerland

Telephone: +41 (0)31 631 33 72

1.9 Data Safety Monitoring Committee

No Data Safety Monitoring Committee is foreseen for this phase 2 clinical trial.

2. ETHICAL AND REGULATORY ASPECTS

2.1 Study registration

This clinical trial will be registered in the SNCTP and in the ClinicalTrials.gov registry prior to the conduct of the trial [1].

2.2 Categorisation of study

The category of this clinical trial is B, since the medicinal product is authorised in Switzerland but in a different indication (thrombosis prophylaxis in orthopaedic surgery) [2, 3].

2.3 Competent Ethics Committee (CEC)

This clinical trial will be submitted for approval to the Competent Ethics Committee of the Kanton Bern (KEK). In case other national or international centres will be opened, the trial will be submitted for approval to the respective CEC as requested. Any study related procedure will be conducted only after written approval from the respective CEC will be available.

2.4 Competent Authorities (CA)

This clinical trial will be submitted for approval to the Competent Authority (Swissmedic). Any study related procedure will be conducted only after written approval from Swissmedic will be available. In case international centres will be included, the trial will be submitted for approval to the respective Compentent Authorities as requested.

2.5 Ethical Conduct of the Study

The study will be carried out in accordance with the protocol and with principles enunciated in the current version of the Declaration of Helsinki, the guidelines of Good Clinical Practice (GCP) issued by ICH, the Swiss Law and Swiss regulatory authority's requirements and any other applicable international law, if applicable. The CEC and regulatory authorities will receive annual safety and interim reports and be informed about study stop/end in agreement with local requirements [2, 4-6].

2.6 Declaration of interest

This clinical trial is supported by a unrestricted financial grant provided by Bayer Pharma AG and Janssen Pharmaceuticals, Inc. Sponsor and investigators declared no other conflict of interest.

2.7 Patient Information and Informed Consent

The investigators will explain to each participant the nature of the study, its purpose, the procedures involved, the expected duration, the potential risks and benefits and any discomfort it may entail. Each participant will be informed that the participation in the study is voluntary and that he/she may withdraw from the study at any time and that withdrawal of consent will not affect his/her subsequent medical assistance and treatment.

The participant must be informed that his/her medical records may be examined by authorised individuals other than their treating physician.

All participants of the study will be provided a participant information sheet and a consent form describing the study and providing sufficient information for participants to make an informed decision about their participation in the study.

The patient information sheet and the consent form will be submitted to the CEC and to the competent authority (as applicable) to be reviewed and approved. The formal consent of a participant, using the approved consent form, must be obtained before the participant is submitted to any study procedure.

The participant should read and consider the statement before signing and dating the informed consent form, and should be given a copy of the signed document. The consent form must also be signed and dated by the investigator (or his designee) and it will be retained as part of the study records.

In case that there is relevant new information regarding the investigational trial product or any relevant adaptation of trial procedures, an amendment of the trial protocol will be submitted to the independent ethics committee and patients will have to re-consent.

2.8 Participant privacy and confidentiality

The investigator affirms and upholds the principle of the participant's right to privacy and that he shall comply with applicable privacy laws. Especially, anonymity of the participants shall be guaranteed when presenting

the data at scientific meetings or publishing them in scientific journals.

Individual subject medical information obtained as a result of this study is considered confidential and disclosure to third parties is prohibited. Subject confidentiality will be further ensured by utilising subject identification code numbers to correspond to treatment data in the computer files.

For data verification purposes, authorised representatives of the Sponsor, a CA (e.g. Swissmedic), or a CEC may require direct access to parts of the medical records relevant to the study, including participants' medical history.

2.9 Early termination of the study

The Sponsor may terminate the study prematurely according to certain circumstances, for example:

- insufficient participant recruitment,
- when the safety of the participants is doubtful or at risk, respectively,
- alterations in accepted clinical practice that make the continuation of a clinical trial unwise, early evidence of benefit or harm of the experimental intervention.

2.10 Protocol amendments

Substantial amendments are only implemented after approval of the CEC and CA respectively.

Under emergency circumstances, deviations from the protocol to protect the rights, safety and well- being of human subjects may proceed without prior approval of the sponsor and the CEC/CA. Such deviations shall be documented and reported to the sponsor and the CEC/CA as soon as possible.

All non-substantial amendments are communicated to the CA as soon as possible if applicable and to the CEC within the Annual Safety Report (ASR).

3. BACKGROUND AND RATIONALE

3.1 Background and Rationale

The prevalence of obesity and morbid obesity is increasing worldwide and is becoming an increasing medical and socioeconomic burden [7]. The rise in morbid obesity led to a significant increase in bariatric surgery driven by the advancements in surgical techniques and use of laparoscopy [8]. Despite key improvements in the perioperative management, there are potential risks, among them venous thromboembolism (VTE) [9]. The incidence of symptomatic deep vein thrombosis (DVT) ranges from 1% - 3%, and the incidence of pulmonary embolism (PE) from 0.3% - 2% [10-12]. The incidence of clinically inapparent venous thromboembolism in bariatric patients is not known, but might be considerably higher, taking into account data reported by Vedovati et al. in patients undergoing laparoscopic surgery for colorectal cancer [13].

Although the overall incidence is low, VTE remains a leading cause of morbidity and mortality after bariatric surgery [14, 15].The American Society for Metabolic and Bariatric Surgery (ASMBS) and the American College of Chest Physicians have recommended in addition to mechanical prophylaxis some methods of VTE prophylaxis with lower extremity compression and some form of pharmacoprophylaxis [16]. However, strategies of adjusted-dose heparins and post discharge anticoagulant prophylaxis of low-molecular-weight heparins as standard treatment were not clearly defined. In addition, there is a lack of randomized controlled trials addressing this topic. Since most post-discharge VTE events occur within the first 28 days after bariatric surgery and the incidence rises within this time-period an extended duration of thromboprophylaxis after hospital discharge should be investigated [17]. Analysis of postdischarge heparin prophylaxis for bariatric surgery is limited to date, but results from Raftopoulus et al. are promising and showed that the 28-day VTE rate was significantly higher in the group that only received in-hospital prophylaxis with enoxaparin compared to the extended pharmacoprophylaxis group [18].

In 2016, the Scientific and Standardization Committee (SSC) of the International Society on Thrombosis and Haemostasis (ISTH) published a guidance document that included a suggestion to not use DOACs in patients with a BMI greater than 40 kg/m^2 or weight exceeding 120 kg due to limited clinical data and concerns regarding the potential effects of body weight extremes on the PK and PD of these agents [19].So far, only limited data are available on the use of direct oral anticoagulants (DOACs) in morbidly obese patients and patients who have undergone bariatric surgery.

Rivaroxaban has been extensively studied in a variety of clinical indications, was generally well tolerated and demonstrated a predictable, dose-dependent pharmacologic profile up to 24 hours after single dose

administration. The anticoagulant has a high oral bioavailability (80-100%) irrespective of food intake, a rapid onset of action and the maximum plasma level is achieved two to four hours after oral administration [20, 21]. One major advantage of DOACs is the fact that there is no need to modify the dosage for extreme body weights up 150 kg [22, 23].

Nevertheless, in these patients anatomic and physiologic changes to the digestive system can influence drug pharmacokinetics, the extent of which may differ over the short and long term. Based on theoretical pharmacological concepts, absorption in patients with a Roux-en-Y-gastric bypass or a sleeve gastrectomy could be impaired due to the fact that parts of the upper GI tract are bypassed or removed, since rivaroxaban is mostly absorbed in the upper gastrointestinal tract including stomach and the small intestine.

However, there was no significantly change of pharmacodynamics and pharmacokinetics in our phase I clinical trial assessing single doses of 10 mg rivaroxaban prior to and after bariatric surgery (see also below at 3.4) [24].

The aim of this study is to investigate the safety and efficacy of rivaroxaban in obese patients undergoing bariatric surgery. Rivaroxaban as an oral anticoagulant could be an attractive option for thromboprophylaxis compared to subcutaneous standard treatment after bariatric surgery. Especially in high-risk patients where an extended duration of thromboprophylaxis after hospital discharge is recommended, an oral therapy would be desirable. The results of this trial will elucidate the clinical applicability of rivaroxaban in bariatric patients and will help to design larger phase III trials in this particular patient population with the final goal of a safe and efficient use of rivaroxaban in morbidly obese patients. Especially in high-risk patients, where an extended duration of thromboprophylaxis after hospital discharge is considered, an oral therapy would be attractive.

3.2 Investigational Product (treatment) and Indication

Rivaroxaban (BAY 59-7939, Xarelto®) is an orally administered, direct Xa inhibitor and is used for the prevention and treatment of thromboembolic disorders. It is rapidly absorbed in the upper gastrointestinal tract. Rivaroxaban is generally well tolerated and demonstrates a predictable, dose- dependent pharmacology profile up to 24 hours after single dose also in obesity patients with no need for dose adjustment irrespective of age, weight and gender [22, 25].

3.3 Preclinical Evidence

Non-clinical data reveal no special hazard for humans based on conventional studies on safety pharmacology, single dose toxicity, phototoxicity, genotoxicity, carcinogenic potential and juvenile toxicity.

Effects observed in repeat-dose toxicity studies were mainly due to the exaggerated pharmacodynamic activity of rivaroxaban. In rats, increased IgG and IgA plasma levels were seen at clinically relevant exposure levels.

In rats, no effects on male or female fertility were seen. Animal studies have shown reproductive toxicity related to the pharmacological mode of action of rivaroxaban (e.g. haemorrhagic complications).

Embryo-foetal toxicity (post-implantation loss, retarded/progressed ossification, hepatic multiple light coloured spots) and an increased incidence of common malformations as well as placental changes were observed at clinically relevant plasma concentrations. The cause of the embryo-foetal toxicity is not known. In the pre- and postnatal study in rats, reduced viability of the offspring was observed at doses that were toxic to the dams [20].

3.4 Clinical Evidence to Date

In multiple Phase I and II studies Rivaroxaban was safe and demonstrated predictable, dose- dependent pharmacokinetics and pharmacodynamics up to 24 hours after single dose application. Rivaroxaban (10 mg) has a high oral bioavailability (80-100%) irrespective of food intake, has a rapid onset of action and the maximum plasma level is achieved two to four hours after oral administration. The oral application of rivaroxaban is approved for the prevention of venous thromboembolism in patients after elective hip and knee surgery in Switzerland (Swissmedic) as well as in the European Union (EMA) and the USA (FDA). In a study by Kubitza et al., Rivaroxaban was well tolerated, C_{max} of rivaroxaban was unaffected in subject >120 kg and the area under the curve (AUC) was unaffected by body weight and gender.

Absorption of rivaroxaban is dependent on the site of drug release in the GI tract. In a study looking at administration of the crushed tablet via a tube, a 29% and 56% decrease in AUC and C_{max} compared to an oral tablet was reported when rivaroxaban granulate was released in the proximal small intestine [26]. It is therefore anticipated that the Roux-en-Y gastric bypass surgery will have some effect on the absorption of rivaroxaban.

However, our phase I clinical investigation showed that the pharmacokinetics and pharmacodynamics of a single 10 mg dose of rivaroxaban did not significantly change after bariatric surgery compared to the investigation in the same patients prior to the surgical intervention [24]. In our clinical trial we could furthermore demonstrated that single doses of rivaroxaban were associated with predictable pharmacodynamics and were well tolerated

in obese patients prior to and 3 days after bariatric surgery [27]. Our findings are in line with a case study reported by Mahlmann et al. where the absorption of rivaroxaban was immediate and not significantly impaired by bariatric surgery of the upper GI tract [28].

3.5 Dose Rationale

Rivaroxaban is approved for the prophylaxis of venous thrombosis after major orthopaedic surgery. In this specific indication a dose of 10 mg rivaroxaban QD is used [29]. In analogy to the approved dose and indication, a dose of 10 mg rivaroxaban QD will be used in this phase 2 clinical trial addressing safety and efficacy of Rivaroxaban in post-bariatric surgery patients.

3.6 Explanation for choice of comparator (or placebo)

No comparator is used in this early clinical trial addressing safety and efficacy of VTE with rivaroxaban in post-bariatric surgery patients.

3.7 Risks / Benefits

Since rivaroxaban is already approved in different indications, there is no general concern regarding its risk profile. The major risk is associated with bleeding. On the other side, ischemic events can be prevented with successful thromboembolic prophylaxis.

Bariatric surgery is performed according to clinical routine. Patients receive the first postoperative dose of rivaroxaban (10 mg) on the first postoperative day. Data of our previous single dose PK/PD study have shown PK/PD effects comparable to non-bariatric postsurgical patients without evidence of a bleeding or a thromboembolic adverse event.

Currently, there is no common recommendation available regarding the duration of VTE prophylaxis in post-bariatric patients. The benefit of this trial will be the generation of new safety and efficacy data regarding the duration of VTE prophylaxis in this specific patient population.

Prophylaxis with rivaroxaban had a significantly higher efficacy in VTE prophylaxis as compared with enoxaparin after hip and knee replacement surgery as was shown in the large RECORD clinical trial programme with similar rates of bleeding [29-32].

With this clinical phase 2 trial that is based on our previous phase 1 PK/PD trial [24], safety and feasibility of an anticoagulation with rivaroxaban will be investigated in bariatric patients. Since to date there is no phase 2 data available for bariatric patients, this trial will be the next step needed to finally introduce a prophylactic anticoagulation with rivaroxaban in bariatric patients.

In case of confirmed safety and efficacy, oral anticoagulation with rivaroxaban will be beneficial for bariatric patients due to the facilitated application in form of tablets compared to daily subcutaneous injections of low-molecular weight heparins (LMWH) after surgery.

Based on the data of our phase 1 PK/PD trial [24] as well as of the available evidence from multiple trials in similar orthopaedic indications [29-32], the risk of the anticoagulation with the investigational product is similar to the standard of care application of LMWH.

With the methodology used in this study (details regarding the sample size calculation see at 11.2), new generalizable information will generated for both study arms.

The most common adverse reactions with Xarelto® are bleeding complications. To minimize the risk for bleeding event, patients with an a priori higher risk for bleeding are excluded from this clinical trial.

The primary safety endpoint will be major bleeding. All adverse events will be collected and processed according to international guidelines and national legislation. Conduct of this clinical trial will be monitored by an independent third party.

Embryo-foetal toxicity (post-implantation loss, retarded/progressed ossification, hepatic multiple light coloured spots) and an increased incidence of common malformations as well as placental changes were observed at clinically relevant plasma concentrations. Therefore, use of a double barrier contraception method during the study is mandatory to minimize the risk for embryo-foetal toxicity.

3.8 Justification of choice of study population

The study population was chosen according to the main objective of this early phase clinical trial, i.e. to investigate the incidence of symptomatic and asymptomatic venous thromboembolism (VTE) in patients with two different post-bariatric anticoagulation strategies.

4. STUDY OBJECTIVES

4.1 Overall Objective

The overall objective of this clinical trial is the investigation of the feasibility of rivaroxaban in the early course after bariatric surgery.

4.2 Primary Objective

To assess the safety and feasibility of VTE prophylaxis with 10 mg rivaroxaban QD for 7 and 28 days, respectively.

4.3 Secondary Objectives

- To compare VTE prophylaxis with 10 mg rivaroxaban QD for 7 and 28 days, respectively, regarding thromboembolic events.
- To assess the postoperative rates of asymptomatic DVT, symptomatic DVT, symptomatic PE, bleeding events, and investigator-reported adverse events (morbidity and mortality).
- To assess the pharmacokinetic effect of 10 mg rivaroxaban after repeated administration in bariatric surgery (subgroup of 32 patients)
- To assess the pharmacodynamic effects of 10 mg rivaroxaban after bariatric surgery (subgroup of 32 patients)

4.4 Safety Objectives

To assess thrombosis- and bleeding-related safety events of rivaroxaban in the early course after bariatric surgery.

5. STUDY OUTCOMES

5.1 Primary Outcome

Primary efficacy outcome is:

Symptomatic or asymptomatic venous thromboembolism (VTE). Composite endpoint consisting of one of the following elements

- any deep vein thrombosis (proximal and distal); suspected symptomatic deep-vein thrombosis will be assessed by ultrasound
- objectively confirmed pulmonary embolism (PE); suspected pulmonary embolism will be confirmed by contrast-enhanced spiral CT.

5.2 Secondary Outcomes

The rationale behind the selection of the secondary outcomes is to investigate the impact of rivaroxaban after bariatric surgery on VTE events and to assess PK and PD parameters after multidose application of rivaroxaban in bariatric patients.

Secondary outcomes are the following parameters:

Clinical outcome variables

- Symptomatic VTE within 28 days after bariatric surgery
- Asymptomatic VTE within 28 days after bariatric surgery
- All-cause mortality within 28 days after bariatric surgery
- Postoperative morbidity (Dindo-Clavien classification) within 28 days after bariatric surgery
- VTE-related death within 28 days postoperatively

Pharmacokinetic parameters (subgroup of 32 rivaroxaban patients, 16 RYGB, 16 SG)

- Rivaroxaban area under plasma concentration curve (AUC)
- Maximum plasma concentration (C_{max}) of rivaroxaban
- Time of maximum plasma concentration (T_{max}) of rivaroxaban
- Rivaroxaban trough plasma concentration (C_{min}) (24 h post last application)

Pharmacodynamic parameters (subgroup of 32 rivaroxaban patients, 16 RYGB, 16 SG)

- Thrombin-antithrombin-complexes (TAT)
- Prothrombin fragment (F1+2)
- D-Dimers
- Thrombin generation assay (TGA)
- Prothrombin time (PT)

5.3 Other Outcomes of Interest

The evaluation of additional outcome variables is not foreseen.

5.4 Safety Outcomes

Primary safety endpoint is the following parameter:

- Major bleeding as defined by ISTH [33].

Secondary safety variables are the following parameters:

- Peripheral thrombosis
- Pulmonary embolism
- Cardiovascular events
- Cerebral ischemia or bleeding
- Drug allergy and drug sensitivity
- Mortality (all cause)
- Mortality (bleeding related)

Additional safety variables:

- Clinically relevant non-major bleeding
- Unexpected haematoma (>25 cm^2)
- Surgical-site bleeding
- Excessive wound haematoma
- Macroscopic haematuria
- Coughing or vomiting blood
- Intra-articular bleeding with trauma
- Any on-treatment bleeding
- Any non-major bleeding
- Haemorrhagic wound complications (the composite of excessive wound haematoma and reported surgical site bleeding)

6. STUDY DESIGN

6.1 General study design and justification of design

This is a randomized, interventional, multicenter phase 2 study with two treatment arms (short vs. extended prophylaxis with rivaroxaban) to assess safety and feasibility of VTE prophylaxis with rivaroxaban performed in the Department of Visceral Surgery at the University Hospital of Bern, Switzerland, Clinic Beau-Site, Visceral Therapy, Switzerland and Department of Surgery, Kantonsspital Baden, Switzerland.

6.2 Methods of minimising bias

6.2.1 Randomisation

Patients will be randomized to one of the two treatment arms. Patients will be stratified according to surgical procedure (RYGB, SG, other bariatric surgery), gender and study centre. The randomization will be performed in 1:1 ratio between the treatment groups. Randomization will be performed via an electronic online randomization system. For the randomization as system with different block sizes will be used.

Selection of bariatric procedure is independent of this study.

6.2.2 Blinding procedures

This is an open label, assessor blinded clinical trial. The compression ultrasound exams of the lower extremities will be performed by vascular ultrasound specialists that are independent from this clinical trial and that are blinded regarding the allocated treatment group. The same will be applicable for the CT scans or lung ventilation/perfusion examinations in case of suspected pulmonary embolism.

6.2.3 Other methods of minimising bias

- Internal and external validity will be addressed in the discussion of the study results.
- A relevant attrition bias is not expected due to the short duration of the study.
- The methods used for the assessment of VTE are validated and widely used in clinical practice. A relevant measurement bias is not expected.
- To minimize a sampling bias, all patients admitted for bariatric surgery will be screened for study participation. However, there might be a sampling bias, since referrals cannot be controlled (less severe cases are probably referred to non-university centers, severe cases to our university center). Due to the participation of a non-university bariatric clinic in this trial sampling bias should be minimized.
- Procedural bias is not expected since patients are randomized to one of the two treatment arms.
- BMI might be a possible confounding factor. A randomized assessment of patients to the different surgical procedure is not feasible due to the fact that the chosen bariatric procedure depends, among other factors, on the BMI (in patients with a higher BMI Sleeve Gastrectomy is the preferred surgical technique).

6.3 Unblinding Procedures (Code break)

Not applicable.

7. STUDY POPULATION

7.1 Eligibility criteria

Participants fulfilling all of the following inclusion criteria are eligible for the study:

- Patient with scheduled elective bariatric surgery or "redo"/revisional surgery after bariatric interventions
  - Roux-en-Y gastric bypass surgery (RYGB) or sleeve gastrectomy (SG)
  - Conversion from SG to RYGB
  - Conversion from gastric banding to SG or RYGB
  - Revision of gastric pouch or gastrojejunal anastomosis
  - Revision of jejuno-jejunostomy

- Patient aged 18 years or older
- BMI ≥ 35 kg/m^2
- Women of child-bearing age: Willingness of using a double barrier contraception method during the study period (up to 4 weeks after end of trial)
- Written, informed consent

The presence of any one of the following exclusion criteria will lead to exclusion of the participant:

- DVT and/or PE in the patient history
- Myocardial infarction, transient ischemic attack or stroke within 6 months of study entry
- Active pathological bleeding
- Uncontrolled hypertension
- Severely impaired hepatic function (cirrhotic patients with Child B or C) or renal function (creatinine clearance <30 mL per min)
- Concomitant treatment with strong CYP3A4 inhibitors (e.g., ketoconazole, itraconazole, lopinavir, ritonavir, indinavir).
- Concomitant treatment with a P-glycoprotein inhibitor and weak or moderate CYP3A4 inhibitor (e.g., erythromycin, azithromycin, diltiazem, verapamil, quinidine, ranolazine, dronedarone, amiodarone, felodipine).
- Concomitant treatment with P-glycoprotein inducers and strong CYP3A4 inducers (e.g. carbamazepine, phenytoin, rifampin).
- Therapy with any other drugs that might affect the study outcome, including anticoagulants, aspirin or other antiplatelet agents, Factor Xa inhibitors other than the study medication, or any other drug affecting coagulation
- Positive pregnancy test, pregnancy or nursing women
- Congenital or acquired bleeding disorder
- Known intolerance of the study medication rivaroxaban

7.2 Recruitment and screening

Obese patients undergoing bariatric surgery will be screened consecutively and enrolled in the study until a total of 260 patients. Screening will be performed during regular outpatient visits in the participating study centres. If the recruitment will be delayed in the above mentioned study centres, additional study centres may be opened.

Patients must meet all of the inclusion criteria and none of the exclusion criteria to be enrolled in the study. All patients will have to give informed consent and agree to the study procedures prior to inclusion to the study. No study-specific procedures (i.e., assessments/treatments not required in clinical practice) may be performed until an eligible patient signed an approved informed consent to participate in the study.

In case of drop outs, additional patients are recruited in order to have a full datasets of 130 patients in each treatment arm. A drop out is defined as a patient who leaves the study prior to the clinical endpoint assessment with compression ultrasound at V7 (study day 28 ±2).

7.3 Assignment to study groups

Patients referred to one of the study centres for bariatric surgery are assessed according to clinical routine standards. The decision to perform bariatric surgery takes into account underlying diseases and is based on the local standard guidelines (in Switzerland the guidelines of the Swiss society for the study of morbid obesity and metabolic disorders [34]).

The type of surgical intervention is determined by an interdisciplinary obesity management team and the decision is taken independent of this clinical trial. The guidelines used for bariatric surgery are based on the International Sleeve Gastrectomy Expert Panel's Consensus Statement of 2012 and the Guidelines for Clinical Application of Laparoscopic Bariatric Surgery of the Society of American Gastrointestinal and Endoscopic Surgeons (SAGES) [35, 36]. SG primarily performed as part of a 2- stage strategy taking into account the elevated perioperative risk in special patient population (advanced age, male gender, preoperative BMI >50 kg/m2, pulmonary hypertension, expected technical difficulty, expected long duration of surgery). Selection of bariatric procedure is independent of this study.

Patients will be randomized to one of the two treatment arms (short versus long prophylaxis with rivaroxaban) and stratified according to the surgical procedure (RYGB, SG, other bariatric surgery), gender and study centre.

For the PK/PD substudy, 16 patients of the SG subset and 16 patients of the RYGB subset will be selected. PK/PD analyses will be performed on patients in the long treatment arm.

7.4 Criteria for withdrawal / discontinuation of participants

7.4.1 Criteria for withdrawal by the patient

Patients may withdraw from the study at any time for any reason, and without prejudice to further treatment.

7.4.2 Criteria for withdrawal/ discontinuation by the investigator

Patients can be withdrawn by the investigator in the following situations:

- Leakage of the anastomosis after bariatric interventions, staple line leakage, major bleeding or thrombotic complication or other relevant medical condition in which continuation of study is not possible or reasonable as assessed by the principal investigator
- Patient non-compliance

8. STUDY INTERVENTION

8.1 Identity of Investigational Product (treatment)

8.1.1 Experimental Intervention (treatment)

The investigational product used in this clinical trial is the factor Xa inhibitor rivaroxaban (Xarelto®). Xarelto® is manufactured by Bayer Pharma AG and Janssen Pharmaceuticals, Inc. and is used in the commercially available oral formulation. In this clinical trial rivaroxaban is used in an off-label indication.

8.1.2 Control Intervention (standard/routine/comparator treatment)

Not applicable.

8.1.3 Packaging, Labelling and Supply (re-supply)

The following information will be included on labels:

a. name, address and telephone number of the sponsor (the main contact for information on the product);
b. the name/identifier and strength/potency, pharmaceutical dosage form, route of administration, quantity of dosage units;
c. the batch and/or code number to identify the contents and packaging operation;
d. a trial reference code allowing identification of the trial, site, investigator and sponsor;
e. the trial subject identification number/treatment number and the visit number;
f. the name of the investigator (see (a));
g. directions for use (reference may be made to a leaflet or other explanatory document intended for the trial subject or person administering the product);
h. “For clinical trial use only”;
i. the storage conditions;
j. period of use (expiry date), in month/year format.

8.1.4 Storage Conditions

Rivaroxaban has to be kept in a secure and locked place at room temperature (below 30℃) according to standard hospital procedures.

8.2 Administration of experimental and control interventions

8.2.1 Experimental Intervention

Study drug will be applied as peroral medication at the indicated time points after bariatric surgery. Arm A: Thromboprophylaxis with rivaroxaban 10 mg QD for 7 days after bariatric surgery.

Arm B: Thromboprophylaxis with rivaroxaban 10 mg QD for 28 days after bariatric surgery.

8.2.2 Control Intervention

Not applicable

8.3 Dose

Rivaroxaban study dose is 10 mg per application.

8.4 Compliance with study intervention

Study drug will be provided by a designated study nurse. Drug compliance will be monitored and documented by the study nurse.

8.5 Data Collection and Follow-up for withdrawn participants

Patients withdrawn by the investigator will be assessed at the time point of the scheduled V7 visit (study day 28 ±2) independent of the time point of study withdrawal.

Patient who withdrew consent will be followed according to local standard of care. This includes routine follow-up visits one and three months after bariatric surgery.

Data and biological samples collected until withdrawal will be further used. Anonymization after withdrawal will not be possible, i.e. data remain coded after withdrawal. Patients will be informed regarding this process (according to ClinO Art. 9) in the informed consent.

8.6 Trial specific preventive measures

In case of a suspected bleeding or thromboembolic event that occurs outside the hospital setting the patient shall immediately contact the study team and seek medical assistance for further medical investigation.

8.7 Concomitant interventions (treatments)

Following concomitant treatments should be avoided during the trial, if clinically possible:
- combined P-glycoprotein and strong CYP3A4 inhibitors and inducers.

Therapy with drugs that might affect the efficacy or safety of the study drug, including anticoagulants, aspirin or other antiplatelet agents, Factor Xa inhibitors other than the study medication, or any other drug affecting coagulation, should be avoided during this trial, if clinically possible.

8.8 Study Drug Accountability

In this clinical trial, commercially available Xarelto® (rivaroxaban) will be labelled and used as study drug. Study drug accountability will be performed by the designated study nurse. Intake of study drug will be recorded in the source documents.

8.9 Return or Destruction of Study Drug

Unused study medication will be returned to the hospital pharmacy and destroyed according to hospital pharmacy standard procedures.

9. STUDY ASSESSMENTS

9.1 Study flow chart

**Study Flow Chart**									
	**Screening**	**Surgery**	**SOT**		**EOT 1**		**EOT 2**		**EOS**
**Study day**	-30 bis -1	0	1		7		28		35
**Study treatment (Rivaroxaban 10 mg qd)**			Short arm						
			Long arm						
PK/PD (subgroup)									
**Aes/SAEs**									

SOT: start of treatment
EOT: end of treatment
EOS: end of study

9.2 Table of study procedures and assessments

Study period	Screening	Surgery	Postoperative																				
Study visit	V0		V1	V2	V3		V4		V5		V6		V7									V8	V9
Study day	-30 bis -1	0	1	2	3	4-7	8±2	9-14	15±2	16-20	21±2	22-27	28±2									V7+1	V7+7
Hosp day		1	2	3	4																		
Time (24 h clock)		8 18+	8	8	8	8	8		8		8		7	8	9	10	11	12	14	16	20	8	
Relative time points for PK/PD (32 patients, 16 RYGB, 16 SG, Inselspital Bern only)**													T -1h	T 0	T 1h	T 2h	T 3h	T 4h	T 6h	T 8h	T 12h	T 24h	
Informed consent	x																						
Medical history	X													x									
Concomitant medication	x													x									
Demographics	x																						
Physical exam incl. weight and height	x													x									
Inclusion/Exclusion-criteria	x																						
Coagulation/hemostasis parameters (INR, Platelets)	x																						
Liver and kidney function	x																						
Beta-HCG	x																						
Bariatric Surgery		x++																					
Study inclusion			x																				
Randomization			x																				
Discharge (earliest point of time)				x																			
Study arms																							
A: Rivaroxaban short arm																							
LMWH in prophylactic dose (e.g. Clexane, Fraxiparine)		x																					
Rivaroxaban 10 mg p.o. QD			x	x	x	x																	
B: Rivaroxaban long arm																							
LMWH in prophylactic dose (e.g. Clexane, Fraxiparine)		x																					
Rivaroxaban 10 mg p.o. QD			x	x	x	x	x	x	x	x	x	x		x									
Duplex sonography																			x				
PK/PD substudy																							
Anti Xa activity**							x		x		x		x		x	x	x	x	x	x	x	x	
Rivaroxaban Concentration**							x		x		x		x		x	x	x	x	x	x	x	x	
Thrombin-Antithrombin-Complex (TAT)**													x		x	x	x	x	x	x	x	x	
Prothrombin-Fragments (F1 + F2)**													x		x	x	x	x	x	x	x	x	
Thrombin Generation**													x		x	x	x	x	x	x	x	x	
D-Dimers	x**		x**				x**		x**		x**		x		x**	x**	x**	x**	x**	x**	x**	x**	
AEs/SAEs		x	x	x	x		x		x		x		x	x	x	x	x	x	x	x	x	x	x*

+1st dose 6–8 h after wound closure or after adequate haemostasis has been achieved, subsequent doses at 8 am

++ Study independent bariatric surgery

* Telephone call

** Analysis PK/PD 32 patients

9.3 Assessments of outcomes

9.3.1 Assessment of primary outcome

Clinical outcome variables

Symptomatic or asymptomatic venous thromboembolism (VTE). Composite endpoint consisting of one of the following elements:

Any deep vein thrombosis of the lower limbs (proximal and distal); suspected symptomatic deep-vein thrombosis and asymptomatic venous thromboembolism will be assessed by compression duplex ultrasound.

Pulmonary embolism (PE); suspected pulmonary embolism will be confirmed by contrast-enhanced spiral CT or ventilation/perfusion szintigraphy.

Primary safety variables

Major bleeding within 28 days after bariatric surgery as defined by the ISTH [37].

Major bleeding is defined as a bleeding event that meets at least one of the following criteria:

- fatal bleeding
- critical bleeding (intracranial, intraocular, intraspinal, pericardial, retroperitoneal, in a nonoperatedjoint, or intramuscular with compartment syndrome)
- clinically overt bleeding (at surgical or extrasurgical site) associated with a decrease in the haemoglobin level of more than 2 g/dL (20 g/L; 1.24 mmol/L) compared with the prerandomisationlevel
- clinically overt bleeding (at surgical or extrasurgical site) leading to transfusion of two or moreunits of whole blood or packed cells
- bleeding located at the surgical site and leading to re-operation or to any unusual medical intervention or procedure for relief (e.g. draining or puncture of an haematoma at the surgicalsite, transfer to an ICU or emergency room)

The occurrence and classification of all components of the composite endpoint and safety outcomes will be centrally adjudicated by an independent and blinded committee of experts.

9.3.2 Assessment of secondary outcomes

Clinical outcome variables

- Symptomatic VTE within 28 days after bariatric surgery
- Asymptomatic VTE within 28 days after bariatric surgery
- All-cause mortality within 28 days after bariatric surgery
- Postoperative morbidity (Dindo-Clavien classification) within 28 days after bariatric surgery
- VTE-related death within 28 days postoperatively

Pharmacokinetic parameters (subgroup of 32 rivaroxaban patients, 16 RYGB, 16 SG)

- Rivaroxaban area under plasma concentration curve (AUC)
- Maximum plasma concentration (C_{max}) of rivaroxaban
- Time of maximum plasma concentration (T_{max}) of rivaroxaban
- Rivaroxaban trough plasma concentration (C_{min}) (24 h post last application)

Pharmacodynamic parameters (subgroup of 32 rivaroxaban patients, 16 RYGB, 16 SG)

- Thrombin-antithrombin-complexes (TAT)
- Prothrombin fragment (F1+2)
- Prothrombin time (PT)
- Thrombin generation assay (TGA)

9.3.3 Assessment of other outcomes of interest

Not applicable.

9.3.4 Assessment of safety outcomes

9.3.4.1 Adverse events

Primary safety variables

– major bleeding (assessment see at 9.3.1)

Secondary safety variable

– peripheral thrombosis (assessment see at 9.3.1)
– pulmonary embolism (assessment see at 9.3.1)
– cardiovascular events
– cerebral ischemia or bleeding
– drug allergy and drug sensitivity
– mortality (all cause)
– mortality (bleeding related)

Additional safety variables

Clinically relevant non-major bleeding within 28 days after bariatric surgery as defined by ISTH [33, 38].

Examples of clinically relevant non-major bleedings are:

– multiple-source bleeding;
– spontaneous hematoma >25 cm^2, or >100 cm^2 if there was a traumatic cause;
– intramuscular hematoma documented by ultrasonography without compartment syndrome;
– excessive wound hematoma not requiring draining or puncture;
– macroscopic hematuria (spontaneous or lasting >24 h if associated with an intervention);
– epistaxis or gingival bleeding that requires tamponade or other medical intervention;
– bleeding after venipuncture for >5 min;
– hemoptysis, hematemesis or spontaneous rectal bleeding requiring endoscopy or other medical intervention.
– Other non-major bleedings
– Other non-major bleedings include other overt bleeding events that do not meet the criteria for major bleeding or clinically relevant non-major bleeding (e.g. epistaxis that does not require medical attention or

change in antithrombotic therapy).

Diseases present prior to the administration of the study intervention will be documented as concomitant diseases as part of the patient history in the CRF

All adverse events will be documented. Adverse events (AEs) are monitored through the follow-up visit V9. Diseases present prior to the administration of the study drug will be documented as concomitant diseases as part of participant history in the CRF.

9.3.5 Procedure in case of confirmed VTE (symptomatic and asymptomatic)

In patients with confirmed VTE, study drug will be stopped and patient will be treated according to current guidelines for the respective VTE [39].

9.3.6 Assessments in participants who prematurely stop the study

Patients with premature study termination will be followed as specified at 7.4.

9.4 Procedures at each visit

9.4.1 Study independent preoperative assessments

Patients who qualify for the study will undergo preoperative work-up according to routine clinical practice. These clinical preoperative investigations are performed to elucidate the causes of obesity and to screen for obesity-related co-morbidities with special attention to those factors that could affect the outcome of bariatric surgery.

This work-up includes the following procedures:

Comprehensive medical history, psycho social history, physical examination and clinical chemistry, hematology and coagulation testing to assess surgical risk according to the SMOB guidelines. Routine laboratory evaluation consists of the following parameters: sodium, potassium, calcium, phosphate, magnesium, glucose, creatinine, uric acid, albumin, transferrin, lipid profile, vitamin

B12, folic acid, HbA1c, TSH, fT3, fT4, cortisol, estradial, LH, FSH, INR, differential blood.

In addition, following assessments are performed:

Gastrointestinal evaluation with an upper endoscopy incl. Helicobacter pylori testing to screen for gastrointestinal haemorrhage or ulcera. Cardiopulmonary risk assessment with sleep apnea screening by a cardiologist. Upper gastrointestinal image series. Abdominal ultrasound in case of symptomatic biliary disease or elevated liver function tests. Endocrine evaluation with ultrasound of the thyroid gland. Clinical nutrition evaluation. Psychosocial-behavioral evaluation by an psychologist [40, 41].

All these assessments are performed as part of the standard of care work-up.

9.4.2 Screening visit V0 (day -30 to day -1)

Study related assessment

Screening visit procedures are specified in the table of study procedures & assessments at 9.2.

As a first step the patient receives detailed information regarding this clinical trial. Patient has to have enough time to reflect all study-associated procedures and duties. No study-associated investigations will be performed before written informed consent is obtained.

Following assessments will be performed as part of this clinical trial:

In female patients pregnancy is excluded with a beta-HCG test in the serum. D-dimers will be measured in the PK/PD subgroup.

Following assessment will be performed independent of this clinical trial:

Study-specific procedures will be performed.

Review of patient history (prior and present diseases, interventions and surgery), demographics (sex, age and ethnicity), history of smoking and use of contraception are documented and a clinical examination is performed to assess skin, lungs, heart, abdomen, circulation and basic neurologic functions. Measurement of weight, size and calculation of BMI takes place. ASA physical status classification system is documented.

A review of all inclusion and exclusion criteria to confirm eligibility will be performed.

9.4.3 Study-independent bariatric surgery and postoperative procedures

Bariatric surgery takes place on study day 0. After bariatric surgery use of elastic stockings as thrombosis prophylaxis and early ambulation are recommended as standard of care in all centres. LMWH is started postoperatively according to standard procedure in the respective study centre.

After the bariatric surgery procedure, patients are allowed to drink water. Patients change their diet from liquid to a soft/normal food within the next days after the bariatric procedure (independent of the type of procedure performed).

9.4.4 Start of treatment visit V1 (day 1)

At V1, inclusion and exclusion parameters will be assessed and patient will be included if all inclusion criteria are met and none of the exclusion criteria is met. Subsequently, patient will be randomized to one of the two treatment arms. Study treatment with rivaroxaban will be started. In case patient is not able to take peroral medication at that point of time according to medical reasons, treatment with LMHW will continue until peroral medication can be provided. Collection of safety data (AE and SAE related data). D-Dimers are sampled and analysed in the subgroup of patients.

9.4.5 Safety visit V2 (day 2)

Collection of safety data (AE and SAE related data).

9.4.6 Safety visit V3 (day 3)

Collection of safety data (AE and SAE related data). Hospital discharge according to routine procedure is independent of this trial.

9.4.7 7 days treatment visit V4 (day 7±1)

End of treatment visit for patients in the short treatment arm. Patients in the long treatment arm will continue treatment through day 28. Collection of PK/PD samples (subgroup only). Collection of safety data (AE and SAE related data).

9.4.8 PK/PD interim visit V5 (day 14±2)

At V5 PK/PD samples are collected (subgroup only) together with AE and SAE data. Study specific blood tubes are sampled at the indicated time points (see Study table of study procedures & assessments at 9.2) at T_{0h} (8 a.m.).

9.4.9 PK/PD interim visit V6 (day 21±2)

At V6 PK/PD samples are collected (subgroup only) together with AE and SAE data. Study specific blood tubes are sampled at the indicated time points (see Study table of study procedures & assessments at 9.2) at T_{0h} (8 a.m.).

9.4.10 Endpoint assessment visit (long arm) V7 (day 28±2)

This study visit takes place during the routine postoperative control in the bariatric outpatients clinic.

End of treatment visit for patients in the long treatment arm. Regular follow-up visit for patients in the short treatment arm.

Review of patient history and concomitant medication.

Physical exam in all patients.

Collection of safety data (AE and SAE related data)

Compression duplex ultrasound assessment of the lower extremities to detect clinical and sub-clinical non VTE (see also 9.4.14).

Collection of PK/PD samples (subgroup only). Study specific blood tubes are sampled at the indicated time points (see “Table of study procedures & assessments” at 9.2) from T_{-1h} to T_{24h}.

9.4.11 24h PK/PD visit V8 (V7+1)

At V8 the 24h PK/PD samples are collected (subgroup only) together with AE and SAE data.

9.4.12 End of study visit V9 (V7+7)

Last study visit is the follow-up visit at day 28±7. Study specific assessment is limited to the collection of safety data (AE and SAE related data).

9.4.13 Laboratory diagnostics

9.4.13.1 Collection of blood samples

Study specific blood tubes are sampled at the indicated study visits and time points (see table of study procedures and assessments at 9.2). Study blood samples are collected throughout the entire study by a trained nurse. Per study time point (as specified in the table of study procedures and assessments at 9.2), 10 ml of citrate blood are withdrawn. In total, 90 ml of blood are withdrawn before and after bariatric surgery, each.

Routine laboratory assessments take place independent of this clinical trial as part of the routine procedure of bariatric patients.

9.4.13.2 Internal analysis of blood samples

Routinely preoperative parameters are analysed in the hospital internal laboratory of each center.

9.4.13.3 External analysis of blood samples

Following parameters are analysed batch-wise in order to avoid analytical variations in the Centre

Hospitalier Universitaire Vaudois (CHUV), Lausanne, Switzerland:

- Anti Xa acivity
- Rivaroxaban concentration
- thrombin-antithrombin-complex (TAT)
- Prothrombin fragments F1+F2
- D-dimers
- thrombin-generation.

Anti Xa activity of heparins is measured using the CE labeled chromogenic anti-FXa assay Biophen Heparin 6 (Hyphen BioMed, Neuilly-sur Oise,France). This is a one stage assay that utilizes endogenous antithrombin. It is an automated kinetic method during which a constant amount of exogenously added bovine FXa is inhibited by anticoagulants in the sample to be tested. Non-inhibited FXa cleaves a FX a-specific chromogenic substrate, producing a yellow signal that is detected at 405 nm.

Rivaroxaban concentration in the patient samples is determined with an anti-FXa assay that is calibrated with commercial samples containing a certified rivaroxaban concentration. This essay has been evaluated against the standard HPLC-MS method and results were comparable [42].

Prothrombin activation fragments 1+2 (F1+F2) and thrombin-antithrombin-complexes (TAT) are measured by a quantitative "sandwich" enzyme immunoassay, according to the protocol of the manufactures (TAT micro and Enzygnost® F1+2 micro, Dade Behring, Marburg, Germany). The absorbance is measured using a microtiter plate reader at 492 nm.

D-dimers concentrations will be determined by ELISA method (Asserachrom, Diagnostica Stago, Asnières, France) [43].

Prothrombin time (PT) is performed using Innovin (Dade Behring), the assay is calibrated with a commercial kit containing 4 defined lyophilized plasmas (DadeBehring), the results are the average of duplicate measurements.

Thrombin generation will be measured by CAT and Fluoroscan Ascent fluorometer (Catalys AG, Wallisellen, Switzerland) using the PPP reagent containing final concentrations of 5 pM tissue factor and 4 µM phospholipids (normal PPP reagent, Diagnostica Stago, Asnières, France) [45]. Assays will be performed by means of Fluoroskan Ascent plate reader and Thrombinoscope© software (Thrombinoscope BV, Maastricht, The Netherlands) as described by the manufacturer. Endogenous thrombin potential (ETP) (nM.min thrombin), representing overall coagulating capacity, lagtime (minutes), peak concentration (nM thrombin) time to peak (minutes) and initial thrombin generation velocity (defined as the rate of the peak and time to peak without lagtime velocity) will be analysed [44].

9.4.13.4 Processing of internal laboratory samples

Routine laboratory samples are processes according to internal standard procedures.

9.4.13.5 Processing and shipment of external non-routine laboratory samples

Processing and shipment of non-routine laboratory samples is specified in appendix 17.2.4

9.4.14 Imaging diagnostics

Study independent imaging diagnostics

Following imaging diagnostic is performed according to clinical routine in the preoperative setting:

- Upper gastrointestinal image series
- Ultrasound of the abdomen and the thyroid gland

Study specific imaging diagnostics

Bilateral compression ultrasonography (CUS) examination of the lower limbs venous system is performed in each patient at visit 7 (28± 2 days) [45].

All sonographers are trained CUS examiners to ensure a high quality of standardized CUS. In patients with suspected or confirmed VTE, video recordings of CUS examinations will be adjudicated centrally. Asymptomatic DVT is defined as VTE detected by screening with ultrasound at V7. Details of reporting see Appendix 17.4.

Clinical assessment of suspected VTE:
Clinical signs and symptoms suggesting VTE will be assessed by bilateral compression ultrasonography (CUS) of the affected venous system.

Clinical assessment of PE:
Clinical signs and symptoms suggesting PE will be confirmed by perfusion/ventilation pulmonary scintigraphy or a spiral computerized tomography. Clinical features (e.g. cyanosis, dyspnoea, tachycardia etc.) will be documented.

10. SAFETY

10.1 Collection and assessment of safety information

10.1.1 Collection of safety information

During the entire duration of the study, all adverse events (AE) and all serious adverse events (SAEs) are collected, fully investigated and documented in source documents and case report forms (CRF). Study duration encompassed the time from when the participant signs the informed consent until the last protocol-specific procedure has been completed, including the safety follow-up visit V9.

10.1.2 Definition and assessment of (serious) adverse events and other safety related events

An **Adverse Event (AE)** is any untoward medical occurrence in a patient or a clinical investigation participant administered a pharmaceutical product and which does not necessarily have a causal relationship with the study procedure. An AE can therefore be any unfavourable and unintended sign (including an abnormal laboratory finding), symptom, or disease temporally associated with the use of a medicinal (investigational) product, whether or not related to the medicinal (investigational) product. ICH E6 1.2

A **Serious Adverse Event (SAE)** is classified as any untoward medical occurrence that:

- results in death,
- is life-threatening,
- requires in-patient hospitalization or prolongation of existing hospitalisation, results in persistent or significant disability/incapacity, or
- is a congenital anomaly/birth defect.

In addition, important medical events that may not be immediately life-threatening or result in death, or require hospitalisation, but may jeopardise the patient or may require intervention to prevent one of the other outcomes listed above should also usually be considered serious. ICH E2A

SAEs will be followed until resolution or stabilisation. Participants with ongoing SAEs at study termination (including safety visit) will be further followed up until recovery or until stabilisation of the disease after termination.

Assessment of Causality

Both Investigator and Sponsor make a causality assessment of the event to the study drug: “Yes” (causal relationship), “No” (no causal relationship).

Unexpected Adverse Drug Reaction

An “unexpected” adverse drug reaction is an adverse reaction, the nature or severity of which is not consistent with the applicable product information. ICH E2A

Suspected Unexpected Serious Adverse Reactions (SUSARs)

The Sponsor evaluates any SAE that has been reported regarding seriousness, causality and expectedness. If the event is related to the investigational product and is both serious and unexpected, it is classified as a SUSAR.

Assessment of Severity

Severity will be assessed by using the criteria specified in the “Common Terminology Criteria for Adverse Events CTCAE Version 4.03”.

10.1.3 Processing of adverse events (AEs) and serious adverse events (SAEs)

Adverse events (AE): Adverse events will be collected for all patients throughout the study period. Adverse Event Documentation: All adverse events occurring after the subject has signed the informed consent must be fully recorded in the subject’s case record form. Documentation must be supported by an entry in the subject’s file. A laboratory test abnormality considered clinically relevant, e.g. causing the subject to withdraw from the trial, requiring treatment or causing apparent clinical manifestations, or judged relevant by the investigator, should be reported as an adverse event. Each event should be described in detail along with start and stop dates, severity, relationship to investigational product, action taken and outcome.

Serious adverse events (SAE): Serious adverse events will be documented according the standard documentation procedures (ICH GCP E6). Reporting of Serious Adverse Events/Pregnancy: Serious adverse events (SAEs), including laboratory test abnormalities fulfilling the definition of serious, after signing the informed consent and during follow-up period must immediately (within 24 hours of the investigator’s awareness) be reported to BAYER PV by fax and/or e-mail (Fax: +41 44 465 8386; e-Mail: drug.safety.switzerland@bayer.ch). A serious adverse event form must also be completed within 24 hours of the investigator awareness and forwarded to BAYER PV by fax and/or e-mail (Fax: +41 44 465 8386; e-Mail: drug.safety.switzerland@bayer.ch). Each serious adverse event must be followed up until resolution or stabilization by submission of updated reports to BAYER PV.

10.1.4 Reporting of serious adverse events (SAEs) and other safety related events

Reporting of SAEs

All SAEs must be reported within a maximum of 24 hours of the investigator’s awareness to the Sponsor of the study. The Sponsor will re-evaluate the SAE and return the form to the site. SAEs resulting in death are reported to the local Ethics Committee (via local Investigator) within 7 days.

Reporting of SUSARs

A SUSAR needs to be reported to the local Ethics Committee (local event via local Investigator) and to Swissmedic for category B studies (via Sponsor) within 7 days, if the event is fatal, or within 15 days (all other events).

Reporting of Safety Signals

All suspected new risks and relevant new aspects of known adverse reactions that require safety- related measures, i.e. so called safety signals, must be reported to the Sponsor within 24 hours. The Sponsor must report the safety signals within 7 days to the local Ethics Committee (local event via local Investigator) and to Swissmedic in case of a category B study.

Reporting and Handling of Pregnancies

Pregnant participants must immediately be withdrawn from the clinical study. Any pregnancy during the treatment phase of the study and within 30 days after discontinuation of study medication must be reported to the Sponsor within 24 hours of the investigator’s awareness. The course and outcome of the pregnancy should be followed up carefully, and any abnormal outcome regarding the mother or the child should be documented and reported.

Periodic reporting of safety

An annual safety report is submitted once a year to the local Ethics Committee via local Investigator and to Swissmedic in case of a category B study via Sponsor.

10.1.5 Follow up of Serious Adverse Events

Any serious adverse event will be followed-up until resolved or in a steady state independent of the study procedures. Loss to follow-up is unlikely since all patients are followed-up as part of the routine post-bariatric procedure.

11. STATISTICAL METHODS

11.1 Hypothesis

This is not a hypothesis driven clinical trial. Aim of the study is the determination of the proportion of 28 day VTE (composite of proximal DVT, PE and VTE-related death) in both treatment arms with a given prevision.

11.2 Determination of Sample Size

This is a safety and feasibility study with the aim of estimating the proportion of 28-day VTE in both treatment arms with a given precision. There will be no formal hypothesis testing between the two treatment arms. All outcomes will be evaluated descriptively in each group presenting estimates with 95% confidence intervals.

We expect a proportion of 28-day VTE of 0.5% and 5% (each including asymptomatic VTE diagnosed with US) in the extended and the short treatment arm, respectively. A sample of 130 patients in each group (260 in total) will result in a two-sided 95% Wilson confidence interval of 0.1-3.7% and 2.4-10.1%, respectively.

In Table 1 the 95 % confidence interval (CI) for several scenarios (combinations of expected proportion = 0.5, 1, 2, 3, 4, 5, 6, 7, 8 % and total sample size = 100, 110, 120, 130, 140, 150 are presented. As expected the length of the CI decreases with larger sample size (i.e. the accuracy of the estimation increases).

Each scenario is evaluated by simulating 1e+05 times the number of events using a binomial distribution (Bin(N,p)). For each simulation the Wilson's score interval is estimated. The presented CI is constructed by taking the mean of the 1e+05 estimates for each scenario.

This was done for all scenarios including the scenarios for which an exact CI could have been calculated (e.g. p = 1, N = 100). In these cases the average expected CI assuming p/N events does not correspond to the exact CI when observing p/N events.

Table 1. Calculation of the 95% Wilson confidence interval (%) varying the number of patients (per arm) and the proportion of expected 28-day VTE in both groups.

	Expected proportion (%)								
Number of patients (each arm)	0.5	1.0	2.0	3.0	4.0	5.0	6.0	7.0	8.0
100	0.1-4.6	0.3-5.4	0.6-6.9	1.1-8.4	1.7-9.7	2.2-11.1	2.9-12.4	3.5-13.7	4.2-14.9
110	0.1-4.2	0.3-5.0	0.7-6.6	1.2-8.0	1.7-9.4	2.3-10.7	3.0-12.0	3.6-13.3	4.3-14.5
120	0.1-4.0	0.3-4.8	0.7-6.3	1.2-7.7	1.8-9.1	2.4-10.4	3.0-11.7	3.7-12.9	4.4-14.2
130	0.1-3.7	0.3-4.5	0.7-6.0	1.2-7.5	1.8-8.8	2.4-10.1	3.1-11.4	3.8-12.7	4.5-13.9
140	0.1-3.5	0.3-4.3	0.7-5.8	1.3-7.2	1.9-8.6	2.5-9.9	3.2-11.2	3.9-12.4	4.6-13.6
150	0.1-3.3	0.3-4.1	0.7-5.6	1.3-7.0	1.9-8.4	2.6-9.7	3.2-11.0	4.0-12.2	4.7-13.4

For the PK/PD analysis no formal sample size estimation has been performed. Samples size has been determined based on common practical aspects for PK/PD analysis in order to have enough information to assess PK/PD parameters with its variations.

11.3 Statistical criteria of termination of trial

Premature termination of the trial based on statistical criteria is not foreseen. In case of serious adverse events that are assessed as related to the study drug, the decision regarding premature trial termination is at the discretion of the Sponsor.

11.4 Planned Analyses

11.4.1 Datasets to be analysed, analysis populations

The full analysis set (FAS) will include all patients that have been randomized to one of the two treatment arms. The SG analysis set (SGAS) will include all patients with successful Sleeve Gastrectomy. The RYGB analysis set (RYAS) will include all patient with successful Roux-en-Y gastric bypass surgery. PK/PD analysis will be performed on a subset of the SG and RY patients (16 patients each). Data will be presented combined as well as for patients with SG and RYGB .

11.4.2 Demographic and baseline characteristics

Patient characteristics will be presented as frequency and percentage for binary variables, mean and standard deviation for continuous, normally distributed variables, and median and interquartile range for continuous, non-normally distributed variables.

11.4.3 Primary Analysis

The primary analysis is an intention-to-treat analysis (ITT), i.e. all patients (FAS) will be analysed in the group they were originally allocated to. The primary binary outcome (i.e. the proportion of 28-day VTE in both treatment arms within 28 days after bariatric surgery) will be evaluated descriptively in each group presenting proportions with 95% Wilson confidence intervals. Additionally, we will compare the primary binary outcome using a Cochran-Mantel-Haenszel-test stratified for the stratification factors used in the randomization. These tests will be exploratory, not confirmatory.

11.4.4 Secondary Analyses

Secondary analyses will be performed per protocol (PP), i.e. only patients that in fact received the study treatment according to the randomization will be analysed. Secondary analyses will be reported for the FAS, the SGAS and the RYAS.

Following secondary outcomes will be analyses:

- Symptomatic VTE within 28 days after bariatric surgery
- Asymptomatic VTE within 28 days after bariatric surgery
- All-cause mortality within 28 days after bariatric surgery
- Postoperative morbidity (Dindo-Clavien classification) within 28 days after bariatric surgery
- VTE-related death within 28 days postoperatively
- PK/PD (subset of 32 patients)

The secondary binary outcomes will be evaluated descriptively in each group presenting proportions with 95% Wilson confidence intervals. Additionally, we will compare the secondary binary outcomes using a Cochran-Mantel-Haenszel-test stratified for the stratification factors used in the randomization. These tests will be exploratory, not confirmatory.

Analysis of pharmacokinetic parameters (subgroup of 32 patients; 16 SB, 16 RYGB)

Rivaroxaban AUC, C_{max}, T_{max} and C_{min} (24h post application) will be calculated and displayed for both surgical procedures combined and separately using the SGAS and the RYAS PK/PD subsets. To calculate and to draw the PK parameters of rivaroxaban, the software application “R” or an equivalent statistical tool will be used. Median as well as the lower and the upper quartile will be presented in a descriptive manner.

Primary analysis of rivaroxaban concentrations will be performed using the data points specified in the study flow chart. For both surgery procedures, the area under the curve (AUC) will serve as primary endpoint. To calculate and draw the pre- and post-surgery area under the curve (AUC) of rivaroxaban, the software application “R” will be used. The data of rivaroxaban AUC is assumed to be lognormally distributed [23]. Therefore, AUC data will be log transformed in order to fulfil normality assumptions. In order to test for equivalence, the equivalence bounds of 0.8 and 1.25 will be used. Analyses will be performed after closure of the database.

The geometric mean ratio (after surgery/before surgery) together with the 95% confidence interval will be estimated for the AUC. The data will be log-transformed in order to better meet normality assumptions. The

confidence interval will be presented graphically in a descriptive manner (exponentiated to bring it back to the original scale).

Analysis of pharmacodynamic parameters (subgroup of 32 patients; 16 SB, 16 RYGB)

Results of PD data (thrombin-antithrombin-complexes (TAT), prothrombin fragment (F1+2), D-Dimers, thrombin generation and prothrombin time) are displayed in tables with median and range and in graphs. PD parameters will be calculated and displayed for combined and separate for both surgical procedures using the SGAS and the RYAS PK/PD subsets.

11.4.5 Interim analyses

No interim analysis will be performed.

11.4.6 Safety analysis

All adverse events and serious adverse events will be assessed regarding relatedness to study drug and presented in tabular form indicating absolute numbers of events and percentages. The safety analysis will be performed on the FAS.

11.4.7 Deviation(s) from the original statistical plan

Deviation from the descriptive statistical analysis plan is not foreseen. In case of deviation from the original statistical plan, this will be specified in the final report.

11.5 Handling of missing data and drop-outs

Missing data will be indicated accordingly and not be replaced. In case of drop-out prior to completion of study visit V8, additional study subjects will be recruited in order to reach the intended number of study participants per group.

12. QUALITY ASSURANCE AND CONTROL

12.1 Data handling and record keeping / archiving

12.1.1 Case Report Forms

Study data is recorded in electronic Case Report Forms (eCRFs; REDCap™) by authorized study personnel. For each enrolled study participant an eCRF is maintained. eCRFs must be kept current to reflect subject status at each phase during the course of study. All data entered in the eCRF must be documented in a source document. Participants must not be identified in the eCRF by name or initials and birth date. Appropriate coded identification must be used. The electronic database will be validated as required by the FDA regulation 21 CFR Guidance for Industry part 11.

The investigator must review all pages within the eCRF for accuracy and consistency with the protocol, and electronically sign the eCRF upon completion.

12.1.2 Specification of source documents

Source data must be available at the site to document the existence of the study participants. Source data must include the original documents relating to the study, as well as the medical treatment and medical history of the participant.

In addition to study specific source data, the electronic patient records of the hospital (incl. laboratory values) may serve as source data. No data is directly recorded in the eCRF (i.e. without source data documentation). All source data must be available at the study centre during the entire duration of the study.

12.1.3 Record keeping / archiving

All study data must be archived for a minimum of 10 years after study termination or premature termination of the clinical trial.

12.2 Data management

12.2.1 Data Management System

Study data is recorded in the worldwide used research electronic data capture application REDCap™. This study database is run by the Clinical Trial Unit (CTU) of the University of Bern. Data is physically stored on servers of the University of Bern that are located in Bern, Switzerland.

12.2.2 Data security, access and back-up

The database servers are kept in a locked, air-conditioned server-room at the University of Bern, Switzerland. Only system and database administrators have access to these servers and back-up tapes. Data back-ups are performed on a daily basis. Back-up tapes are stored in a safe, different location. Authorized users have access to the database using a login and a personal password. Access is restricted depending on the role of the study team member (role concept: principal investigator, investigator, study nurse, monitor, statistician, administrator).

12.2.3 Analysis and archiving

Data are extracted electronically from the electronic data capture system REDCap™ after closure of the database for statistical analysis. Data for statistical analysis is processed on access controlled computers of the University Hospital Inselspital, Bern, Switzerland.

All study data must be archived for a minimum of 10 years after study termination or premature termination of the clinical trial.

12.2.4 Electronic and central data validation

Data is validated upon entry in the electronic data capture system (range and consistency checks) and will be validated centrally by the principal investigator or his deputy. In addition, an on-site monitoring will be performed (see below).

12.3 Monitoring

On-site Monitoring will be performed by the Clinical Trial Unit Bern, Finkenhubelweg 11, Bern, Switzerland or an equivalent service provider. Monitoring details (number of visits and data/documents to be monitored) will be specified in a monitoring plan before the study initiation visit.

Source data/documents must be kept accessible to monitors and questions answered during regular monitoring visits.

12.4 Audits and Inspections

Study documentation and the source data/documents are accessible to auditors/inspectors (CEC and CA) and questions are answered during inspections. All involved parties must keep the participant data strictly confidential.

12.5 Confidentiality, Data Protection

Study personnel has access to study related documentation and study documents (source documents and eCRF) according to his/her specific role. Access to source documents will be permitted for purposes of monitoring, audits and inspections. All involved parties must keep the participant data strictly confidential. Data protection will be based on local and national guidelines and applicable local and national law.

12.6 Storage of biological material and related health data

Biological material will be stored for a maximum duration of 10 years. Study samples are only stored with the participants consent independent from the study. In case of lacking consent regarding biobanking of study samples, all biological material will be destroyed after termination of the study.

13. PUBLICATION AND DISSEMINATION POLICY

The results of the study will be published in a peer reviewed scientific medical journal and may be presented

via oral communications or poster presentations during national and international meetings.

Co-authorship on any of the publications will be based on conceptual contribution to the study according to the criteria of the International Committee of Medical Journal Editors.

14. FUNDING AND SUPPORT

14.1 Funding

This clinical study is supported by an unrestricted grant from Bayer Pharma AG and Janssen Pharmaceuticals, Inc.

14.2 Other Support

Other support for this clinical trial is not foreseen.

15. INSURANCE

Insurance coverage will be provided by the Sponsor for all study centres. A copy of the insurance certificate is filed in the investigator site file/trial master file.

16. REFERENCES

1. *WHO, International Clinical Trials Registry Platform (ICTRP). (http://www.who.int/ictrp/en/).*
2. *Humanforschungsgesetz (HFG) vom 30. September 2011. (http://www.bag.admin.ch/themen/medizin/00701/00702/07558/index.html?lang=de).*
3. *Verordnung über klinische Versuche in der Humanforschung (KlinV) vom 20. September 2013. (http://www.bag.admin.ch/themen/medizin/00701/00702/12310/index.html?lang=de).*
4. *Declaration of Helsinki, Version October 2013, (http://www.wma.net/en/30publications/10policies/b3/index.html).*
5. *International Conference on Harmonization (ICH, 1997) E8 Guideline: General Considerations for Clinical Trials http://www.ich.org/fileadmin/Public_Web_Site/ICH_Products/Guidelines/Efficacy/E8/Step4/E8_Guideline.pdf.*
6. *Heilmittelgesetz (HMG), vom 15. Dezember 2000. (http://www.admin.ch/ch/d/sr/8/812.21.de.pdf).*
7. WHO., *WHO. WHO obesity and overweight fact sheet no 311. .*
8. Nguyen, N.T., et al., *Trends in use of bariatric surgery, 2003-2008.* J Am Coll Surg, 2011. **213**(2): p. 261-6.
9. Aminian, A., et al., *How safe is metabolic/diabetes surgery?* Diabetes Obes Metab, 2015. **17**(2): p. 198-201.
10. Quebbemann, B., M. Akhondzadeh, and R. Dallal, *Continuous intravenous heparin infusion prevents peri-operative thromboembolic events in bariatric surgery patients.* Obes Surg, 2005. **15**(9): p. 1221-4.
11. Escalante-Tattersfield, T., et al., *Incidence of deep vein thrombosis in morbidly obese patients undergoing laparoscopic Roux-en-Y gastric bypass.* Surg Obes Relat Dis, 2008. **4**(2): p. 126-30.
12. Kardys, C.M., et al., *Safety and efficacy of intravascular ultrasound-guided inferior vena cava filter in super obese bariatric patients.* Surg Obes Relat Dis, 2008. **4**(1): p. 50-4.
13. Vedovati, M.C., et al., *A randomized study on 1-week versus 4-week prophylaxis for venous thromboembolism after laparoscopic surgery for colorectal cancer.* Ann Surg, 2014. **259**(4): p. 665-9.
14. Morino, M., et al., *Mortality after bariatric surgery: analysis of 13,871 morbidly obese patients from a national registry.* Ann Surg, 2007. **246**(6): p. 1002-7; discussion 1007-9.
15. Hamad, G.G. and D. Bergqvist, *Venous thromboembolism in bariatric surgery patients: an update of risk and prevention.* Surg Obes Relat Dis, 2007. **3**(1): p. 97-102.
16. American Society for, M. and C. Bariatric Surgery Clinical Issues, *ASMBS updated position statement on prophylactic measures to reduce the risk of venous thromboembolism in bariatric surgery patients.* Surg Obes Relat Dis, 2013. **9**(4): p. 493-7.
17. Winegar, D.A., et al., *Venous thromboembolism after bariatric surgery performed by Bariatric Surgery Center of Excellence Participants: analysis of the Bariatric Outcomes Longitudinal Database.* Surg Obes Relat Dis, 2011. **7**(2): p. 181-8.
18. Raftopoulos, I., et al., *The effect of extended post-discharge chemical thromboprophylaxis on venous thromboembolism rates after bariatric surgery: a prospective comparison trial.* Surg Endosc, 2008. **22**(11): p. 2384-91.
19. Martin, K., et al., *Use of the direct oral anticoagulants in obese patients: guidance from the SSC of the ISTH.* J Thromb Haemost, 2016. **14**(6): p. 1308-13.
20. *Bayer Pharma AG. Xarelto® (rivaroxaban) summary of product characteristics. http://www.ema.europa.eu/docs/en_GB/document_library/EPAR_-_Product_Information/human/000944/WC500057108.pdf (accessed January 10, 2017). .*
21. Kubitza, D., et al., *Safety, pharmacodynamics, and pharmacokinetics of BAY 59-7939--an oral, direct Factor Xa inhibitor--after multiple dosing in healthy male subjects.* Eur J Clin Pharmacol, 2005. **61**(12): p. 873-80.
22. Kubitza, D., et al., *Body weight has limited influence on the safety, tolerability, pharmacokinetics, or pharmacodynamics of rivaroxaban (BAY 59-7939) in healthy subjects.* J Clin Pharmacol, 2007. **47**(2): p. 218-26.
23. Kubitza, D., et al., *Safety, pharmacodynamics, and pharmacokinetics of single doses of BAY 59-*

7939, an oral, direct factor Xa inhibitor. Clin Pharmacol Ther, 2005. **78**(4): p. 412-21.

24. Kroll, D., et al., *Pharmacokinetics and pharmacodynamics of single doses of Rivaroxaban in obese patients before and after bariatric surgery.* Br J Clin Pharmacol, 2017.
25. Perzborn, E., et al., *Rivaroxaban: a new oral factor Xa inhibitor.* Arterioscler Thromb Vasc Biol. **30**(3): p. 376-81.
26. Moore, K.T., Krook, M. A. Vaidyanathan, S., Sarich, T.C., Damaraju, C.V., Fields, L.E., *Rivaroxaban crushed tablet suspension characteristics and relative bioavailability in healthy adults when administered orally or via nasogastric tube.* Clinical Pharmacology in Drug Development, 2014. **Volume 3**(Issue 4): p. 321-327.
27. Moore, K.T. and D. Kroll, *Influences of Obesity and Bariatric Surgery on the Clinical and Pharmacologic Profile of Rivaroxaban.* Am J Med, 2017. **130**(9): p. 1024-1032.
28. Mahlmann, A., S. Gehrisch, and J. Beyer-Westendorf, *Pharmacokinetics of rivaroxaban after bariatric surgery: a case report.* J Thromb Thrombolysis, 2013.
29. Turpie, A.G., et al., *Rivaroxaban versus enoxaparin for thromboprophylaxis after total knee arthroplasty (RECORD4): a randomised trial.* Lancet, 2009. **373**(9676): p. 1673-80.
30. Eriksson, B.I., et al., *Rivaroxaban versus enoxaparin for thromboprophylaxis after hip arthroplasty.* N Engl J Med, 2008. **358**(26): p. 2765-75.
31. Lassen, M.R., et al., *Rivaroxaban versus enoxaparin for thromboprophylaxis after total knee arthroplasty.* N Engl J Med, 2008. **358**(26): p. 2776-86.
32. Kakkar, A.K., et al., *Extended duration rivaroxaban versus short-term enoxaparin for the prevention of venous thromboembolism after total hip arthroplasty: a double-blind, randomised controlled trial.* Lancet, 2008. **372**(9632): p. 31-9.
33. Schulman, S., et al., *Definition of major bleeding in clinical investigations of antihemostatic medicinal products in non-surgical patients.* J Thromb Haemost, 2005. **3**(4): p. 692-4.
34. *Swiss Society for the Study of Morbid Obesity and Metabolic Disorders (SMOB), Richtlinien zur operativen Behandlung von Übergewicht (www.smob.ch).* 2014.
35. Rosenthal, R.J., et al., *International Sleeve Gastrectomy Expert Panel Consensus Statement: best practice guidelines based on experience of >12,000 cases.* Surg Obes Relat Dis, 2012. **8**(1): p. 8-19.
36. *SAGES guideline for clinical application of laparoscopic bariatric surgery.* Surg Obes Relat Dis, 2009. **5**(3): p. 387-405.
37. Schulman, S., et al., *Definition of major bleeding in clinical investigations of antihemostatic medicinal products in surgical patients.* J Thromb Haemost, 2010. **8**(1): p. 202-4.
38. Ruff, C.T., et al., *Evaluation of the novel factor Xa inhibitor edoxaban compared with warfarin in patients with atrial fibrillation: design and rationale for the Effective aNticoaGulation with factor xA next GEneration in Atrial Fibrillation-Thrombolysis In Myocardial Infarction study 48 (ENGAGE AF-TIMI 48).* Am Heart J, 2010. **160**(4): p. 635-41.
39. *AWMF Leilinien. Diagnostik und Therapie der Venenthrombose und der Lungenembolie. http://www.awmf.org/uploads/tx_szleitlinien/065-002l_S2k_VTE_2016-01.pdf.*
40. Mechanick, J.I., et al., *American Association of Clinical Endocrinologists, The Obesity Society, and American Society for Metabolic & Bariatric Surgery Medical Guidelines for Clinical Practice for the perioperative nutritional, metabolic, and nonsurgical support of the bariatric surgery patient.* Surg Obes Relat Dis, 2008. **4**(5 Suppl): p. S109-84.
41. Fried, M., et al., *Interdisciplinary European guidelines for surgery for severe (morbid) obesity.* Obes Surg, 2007. **17**(2): p. 260-70.
42. Asmis, L.M., et al., *Rivaroxaban: Quantification by anti-FXa assay and influence on coagulation tests: a study in 9 Swiss laboratories.* Thromb Res, 2012. **129**(4): p. 492-8.
43. Chilver-Stainer, L., B. Lammle, and L. Alberio, *Titre of anti-heparin/PF4-antibodies and extent of in vivo activation of the coagulation and fibrinolytic systems.* Thromb Haemost, 2004. **91**(2): p. 276-82.
44. Hemker, H.C., et al., *Thrombin generation, a function test of the haemostatic-thrombotic system.* Thromb Haemost, 2006. **96**(5): p. 553-61.
45. Lensing, A.W., et al., *Detection of deep-vein thrombosis by real-time B-mode ultrasonography.* N Engl J Med, 1989. **320**(6): p. 342-5.

17. APPENDICES

17.1 Summary of product characteristics

Xarelto 10 mg / 15 mg / 20 mg film-coated tablets
(Abbreviated version)
This medicinal product is subject to additional monitoring.

Composition:
Active ingredient: 10 mg / 15 mg / 20 mg rivaroxaban.
Excipients: Microcrystalline cellulose, croscarmellose sodium, lactose monohydrate, hypromellose, sodium laurilsulfate, magnesium stearate, macrogol 3350, titanium dioxide (E171), iron oxide red (E172).

Indications:
10 mg: Prevention of venous thromboembolism (VTE) in adult patients undergoing elective hip or knee replacement surgery. *15 mg/20 mg:* Prevention of stroke and systemic embolism in adult patients with non-valvular atrial fibrillation with one or more risk factors, such as congestive heart failure, hypertension, age ≥ 75 years, diabetes mellitus, prior stroke or transient ischaemic attack. Treatment of deep vein thrombosis (DVT) and pulmonary embolism (PE), and prevention of recurrent DVT and PE in adults.

Contraindications:
Hypersensitivity to the active substance or any of the excipients; active clinically significant bleeding; lesion or condition if considered a significant risk for major bleeding; concomitant treatment with any other anticoagulants except under the circumstances of switching therapy to or from rivaroxaban or when unfractionated heparin is given at doses necessary to maintain an open central venous or arterial catheter; hepatic disease associated with coagulopathy and clinically relevant bleeding risk including cirrhotic patients with Child Pugh B and C; pregnancy and breast feeding.

Warnings and Precautions:
Clinical surveillance in line with anticoagulation practice is recommended throughout treatment. Xarelto should be discontinued if severe haemorrhage occurs. Increasing age may increase haemorrhagic risk. *Not recommended:* in patients with severe renal impairment (creatinine clearance <15 ml/min); in patients receiving concomitant systemic treatment with strong concurrent CYP3A4- and P-gp-inhibitors, i.e. azole-antimycotics or HIV protease inhibitors; in patients with increased bleeding risk; in patients receiving concomitant treatment with strong CYP3A4 inducers unless the patient is closely observed for signs and symptoms of thrombosis; *not recommended due to lack of data:* in patients below 18 years of age, in patients concomitantly treated with dronedarone. 10 mg add: in patients undergoing hip fracture surgery;

15 mg / 20 mg add: in patients with prosthetic heart valves, in patients with PE who are haemodynamically unstable or may receive thrombolysis or pulmonary embolectomy. *Use with caution:* in conditions with increased risk of haemorrhage; in patients with severe renal impairment (creatinine clearance 15 - 29 ml/min) or with renal impairment concomitantly receiving other medicinal products which increase rivaroxaban plasma concentrations; in patients treated concomitantly with medicinal products affecting haemostasis. 10 mg add: when neuraxial anaesthesia or spinal/epidural puncture is employed; 15 mg / 20 mg add: specific dose recommendations apply for patients with moderate to severe renal impairment and in case of

DVT/PE-patients only if the patient's assessed risk for bleeding outweighs the risk for recurrent DVT/PE. In patients at risk of ulcerative gastrointestinal disease prophylactic treatment may be considered. Although treatment with rivaroxaban does not require routine monitoring of exposure, rivaroxaban levels measured with a calibrated quantitative anti-Factor Xa assay may be useful in exceptional situations. Xarelto contains lactose.

Undesirable effects:
Common: anaemia, dizziness, headache, eye haemorrhage, hypotension, haematoma, epistaxis, haemoptysis, gingival bleeding, gastrointestinal tract haemorrhage, gastrointestinal and abdominal pains, dyspepsia, nausea, constipation, diarrhoea, vomiting, pruritus, rash, ecchymosis, cutaneous and subcutaneous haemorrhage, pain in extremity, urogenital tract haemorrhage (menorrhagia *very common* in women < 55 years treated for DVT, PE or prevention of recurrence), renal impairment, fever, peripheral oedema, decreased general strength and energy, increase in transaminases, post-procedural haemorrhage, contusion, wound secretion.
Uncommon: thrombocythemia, allergic reaction, dermatitis allergic, cerebral and intracranial haemorrhage, syncope, tachycardia, dry mouth, hepatic function abnormal, urticaria, haemarthrosis, feeling unwell, increases in: bilirubin, blood alkaline phosphatase, LDH, lipase, amylase, GGT.
Rare: jaundice, muscle haemorrhage, localised oedema, bilirubin conjugated increased, vascular pseudoaneurysm.
Frequency not known: compartment syndrome or (acute) renal failure secondary to a bleeding, angioedema and allergic oedema (*uncommon* in pooled phase III trials).

Classification for supply:
Medicinal product subject to medical prescription.

Marketing Authorisation Holder:
Bayer Pharma AG, D-13342 Berlin, Germany

Further information available from:
medinfo@bayerhealthcare.com

Version:
EU/2

17.2 Processing of study blood samples

17.2.1 Processing of routine in-house blood samples

Blood tubes for test that are performed in-house will be sampled and processed according to in-house standard procedures.

17.2.2 Processing of non-routine blood samples

Platelet poor plasma (PPP) containing less than 10’000 platelets per ml will be prepared by centrifugation at 1’500 g for 10 minutes each at 20°C. PPP samples will be aliquoted (1 ml polypropylene screw-cap tubes), snap-frozen and stored at -80°C until testing. Centrifugation of blood samples has to be performed within 24 hours.

17.2.3 Labelling of non-routine blood samples

Each tube has to be labelled with the study subject number, the visit number, date and the exact point of time the blood has been drawn.

17.2.4 Shipment of non-routine blood samples to the CHUV, Lausanne

After V8 visit of the last study subject, samples are sent batch-wise on dry ice to Lausanne for further processing and analysis.

17.3 Packaging, Labelling and Supply (re-supply)

The following information will be included on labels:

a. Name, address and telephone number of the sponsor (the main contact for information on the product): Dr. med. Dino Kröll, Inselspital, 3010 Bern, Tel. 031 632 24 03
b. Name/identifier and strength/potency, pharmaceutical dosage form, route of administration, quantity of dosage units; Xarelto (Rivaroxaban) Tbl. 10 mg, oral, application of one dosage unit
c. Batch and/or code number to identify the contents and packaging operation: To be specified
d. Trial reference code UVCMBS001; site Bern; sponsor Dr. med. Dino Kröll
e. Trial subject identification number xx; treatment number yy and the visit number zz
f. Name of the investigator: see (a)
g. Directions for use (explanatory document for the person administering the product): use according to "Fachinformation Xarelto, Compendium"
h. "For clinical trial use only";
i. Storage conditions: room temperature (below 30℃)
j. Period of use (expiry date): month/year

17.4 Standardized Duplex Ultrasound Reporting

Exam **TVT screening**

Date<br>Study number<br>patient ID

			comment
V. cava inferior			
	right side	left side	comment
V. iliaca commjnis			
V. iliaca externa			
V. ilicac interna			
V. femoralis communis			
V. femoralis profunda			
V. femoralis supcerficialis prox.			
V. femoralis superficialis distal			
V(v) popliteae			
Vv tibiales anteriores			
Vv tibiales posteriores			
Vv fiburlares			
Crural muscle veins			
V. saphena magna			
V. saphena parva			

Assessment	
