# Supplementary material for: Efficacy and Safety of Rivaroxaban for Postoperative Thromboprophylaxis in Patients After Bariatric Surgery: A Randomized Clinical Trial
Source: JAMA Netw Open. 2023 May 25;6(5):e2315241. doi: 10.1001/jamanetworkopen.2023.15241 (PMC10214035; doi:10.1001/jamanetworkopen.2023.15241)
Supplement: Supplement 2. — eMethods 1. In- and Exclusion Criteria eMethods 2. Standard Postoperative Thromboprophylaxis and Nutrition Management eMethods 3. Statistical Analysis eDiscussion 1. Subgroups eDiscussion 2. Risk-Adjusted Approaches for Duration of Thromboembolic Prophylaxis eDiscussion 3. Statement of the ISTH eTable 1. Clinically Non-relevant Bleedings eTable 2. Postoperative Complications & Adverse Events eTable 3. Overview of Secondary Outcome in Patients Who Underwent RYGB, SG and RS (Safety Population) eFigure 1. Cumulative Event Rate of Symptomatic and Asymptomatic VTE (iMTT Data Set, n=257) eFigure 2. Cumulative Event Rate of Any Major and Clinically Relevant Non-Major Bleeding (Safety Population, n=269) [file jamanetwopen-e2315241-s002.pdf]

# Supplementary Online Content

Kröll D, Nett PC, Rommers N, et al. Efficacy and safety of rivaroxaban for postoperative thromboprophylaxis in patients after bariatric surgery: a randomized clinical trial. *JAMA Netw Open*. 2023;6(5):e2315241. doi:10.1001/jamanetworkopen.2023.15241

**eMethods 1.** In- and Exclusion Criteria
**eMethods 2.** Standard Postoperative Thromboprophylaxis and Nutrition Management
**eMethods 3.** Statistical Analysis
**eDiscussion 1.** Subgroups
**eDiscussion 2.** Risk-Adjusted Approaches for Duration of Thromboembolic Prophylaxis
**eDiscussion 3.** Statement of the ISTH
**eTable 1.** Clinically Non-relevant Bleedings
**eTable 2.** Postoperative Complications & Adverse Events
**eTable 3.** Overview of Secondary Outcome in Patients Who Underwent RYGB, SG and RS (Safety Population)
**eFigure 1.** Cumulative Event Rate of Symptomatic and Asymptomatic VTE (iMTT Data Set, n=257)
**eFigure 2.** Cumulative Event Rate of Any Major and Clinically Relevant Non-Major Bleeding (Safety Population, n=269)

This supplementary material has been provided by the authors to give readers additional information about their work.

© 2023 Kröll D et al. *JAMA Network Open.*

## eMethods 1: In- and exclusion criteria

Additional exclusion criteria were uncontrolled (severe) hypertension and any condition contraindicating the use of rivaroxaban, including severely impaired hepatic or renal function (creatinine clearance <30 mL per min). Other ineligibility criteria included the concomitant treatment with combined P-glycoprotein and CYP3A4 inhibitors, P-glycoprotein inducers or strong CYP3A4 inducers (e.g., carbamazepine, phenytoin or rifampin), aspirin, other antiplatelet agents or Factor Xa inhibitors other than study medication.

## eMethods 2: Standard postoperative thromboprophylaxis and nutrition management

A single prophylactic dose of low molecular weight heparin (LMWH) was given 6-8 hours after surgery according to standard procedure in the respective study centre. LMWH was stopped at randomization. As non-pharmacologic thrombosis prophylaxis early mobilization and intermittent pneumatic compression was used according to local standard of care. After the bariatric surgery procedure, patients were allowed to drink 500 ml water during the first 24 h with unlimited liquid intake thereafter. In parallel, a low-sugar clear liquid meal program was initiated and maintained for 14 days.

## eMethods 3: Statistical Analysis

To calculate the sample size, different scenarios were evaluated by simulating hundred thousand times the number of events using a binomial distribution with an expected proportion of 28-day VTE of 0.5% and 5% (including asymptomatic VTE) in the extended and the short prophylaxis group, respectively. A sample of 130 patients in each group (260 in total) would result in a two-sided 95% Wilson confidence interval of 0.1-3.7% and 2.4-10.1% in the extended and short prophylaxis group, respectively.

Secondary outcome measures were reported in the per protocol population, consisting of all patients who took the study medication as prescribed. The number of events was described per treatment group for all outcomes of interest, together with the proportion and corresponding 95% Wilson confidence interval (CI). To calculate the CI intervals where the number of events was 0, a continuity corrections was used.

Between-group differences for the occurrence of any bleedings and any postoperative complications were estimated using the Cochran-Mantel-Haenszel test for weighted risk

© 2023 Kröll D et al. *JAMA Network Open.*

reduction (stratified by surgery type). The absolute risk difference were presented with corresponding two-sided 95% CI.

## eDiscussion 1: Subgroups

So far, most bariatric trials investigated the outcome of thromboprophylaxis in RYGB patients, whereas data for SG patients, currently the most frequently performed surgical procedure worldwide, and for RS patients are lacking. This trial did not show any relevant difference between different bariatric surgery procedures (with the limitation that the number of patients with revisional surgery was quite low).

## eDiscussion 2: Risk-adjusted approaches for duration of thromboembolic prophylaxis

Some authors used risk-adjusted approaches to VTE prophylaxis, including parameters like BMI, age, sex, history of VTE, non-gastric band surgery, dyspnoea, sleep apnea and length of hospital stay in their risk assessment to guide the use of prophylactic anticoagulation after hospital discharge. However, such risk adapted algorithms are not well established, and randomized controlled trials to determine the optimal duration of TVE prophylaxis after bariatric surgery are missing.


- Aminian A, Andalib A, Khorgami Z, Cetin D, Burguera B, Bartholomew J, et al. Who Should Get Extended Thromboprophylaxis After Bariatric Surgery?: A Risk Assessment Tool to Guide Indications for Post-discharge Pharmacoprophylaxis. Annals of surgery. 2017;265(1):143-50.
- Finks JF, English WJ, Carlin AM, Krause KR, Share DA, Banerjee M, et al. Predicting risk for venous thromboembolism with bariatric surgery: results from the Michigan Bariatric Surgery Collaborative. Annals of surgery. 2012;255(6):1100-4.
- Pannucci CJ, Swistun L, MacDonald JK, Henke PK, Brooke BS. Individualized Venous Thromboembolism Risk Stratification Using the 2005 Caprini Score to Identify the Benefits and Harms of Chemoprophylaxis in Surgical Patients: A Meta-analysis. Annals of surgery. 2017;265(6):1094-103.
- Dang JT, Switzer N, Delisle M, Laffin M, Gill R, Birch DW, et al. Predicting venous thromboembolism following laparoscopic bariatric surgery: development of the BariClot tool using the MBSAQIP database. Surgical endoscopy. 2019;33(3):821-31.


## eDiscussion 3: Statement of the ISTH

In the latest recommendation, the Scientific and Standardization Committee of the International Society on Thrombosis and Haemostasis (ISTH) supports for the first time the use of rivaroxaban in patients with high BMI and bodyweight due to emerging data from multiple retrospective

analyses from phase III studies. However, in the acute postoperative setting, the use of DOAC to prevent VTE is still not recommended. ISTH suggests not to use DOAC in patients with a body weight >120 kg or a BMI>40 kg/m2 due to limited clinical data.


- Martin KA, Beyer-Westendorf J, Davidson BL, Huisman MV, Sandset PM, Moll S. Use of direct oral anticoagulants in patients with obesity for treatment and prevention of venous thromboembolism: Updated communication from the ISTH SSC Subcommittee on Control of Anticoagulation. Journal of thrombosis and haemostasis : JTH. 2021;19(8):1874-82.
- Moore KT, Kröll D. Influences of Obesity and Bariatric Surgery on the Clinical and Pharmacologic Profile of Rivaroxaban. Am J Med. 2017;130(9):1024-32.
- Nasser MF, Jabri A, Gandhi S, Rader F. Oral Anticoagulant Use in Morbid Obesity and Post Bariatric Surgery: A Review. Am J Med. 2021;134(12):1465-75.
- Martin K, Beyer-Westendorf J, Davidson BL, Huisman MV, Sandset PM, Moll S. Use of the direct oral anticoagulants in obese patients: guidance from the SSC of the ISTH. Journal of thrombosis and haemostasis : JTH. 2016;14(6):1308-13.

## eTable 1: Clinically non-relevant bleedings

| | Rivaroxaban 7 days [n (%)] | Rivaroxaban 28 days [n (%)] |
|---|---|---|
| n | 3 | 7 |
| Epistaxis | 1 (33.3) | 2 (28.6) |
| Few ml of blood in Douglas space probably from minor intra-operative bleeding | 1 (33.3) | 0 (0.0) |
| Gingival bleeding | 0 (0.0) | 1 (14.3) |
| Hypermenorrhea | 1 (33.3) | 2 (28.6) |
| Macrohematuria, cystitis | 0 (0.0) | 1 (14.3) |
| Spotting | 0 (0.0) | 1 (14.3) |

## eTable 2: Postoperative complications & adverse events

| Adverse events | Overall (n=269) | Rivaroxaban 7 days (n=134) | Rivaroxaban 28 days (n=135) | p-values* |
|---|---|---|---|---|
| Any adverse event [n] | 72 | 28 | 44 | 0.08 |
| Patients with adverse events [n (% of all patients)] | 58 (21.6) | 23 (17.2) | 35 (25.9) | 0.18 |
| Serious adverse events [n (% of all AEs)] | 19 (26.4) | 6 (21.4) | 13 (29.5) | 0.70 |
| Patients with serious adverse events [n (% of all patients)] | 18 (6.7) | 6 (4.5) | 12 (8.9) | 0.80 |
| Adverse event leading to medication discontinuation [n (% of all AEs)] | 12 (16.7) | 3 (10.7) | 9 (20.5) | 0.53 |
| Patients with adverse events leading to medication discontinuation [n (% of all patients)] | 12 (4.5) | 3 (2.2) | 9 (6.7) | 0.50 |

*post-hoc explorative

© 2023 Kröll D et al. *JAMA Network Open.*

**eTable 3: Overview of secondary outcome in patients who underwent RYGB, SG and RS (safety population)**

| | Overall | | Rivaroxaban 7 days | | Rivaroxaban 28 days | |
|---|---|---|---|---|---|---|
| | n | proportion (%) [95% CI] | n | proportion (%) [95% CI] | n | proportion (%) [95% CI] |
| **RYGB** | n = 132 | | n = 67 | | n = 65 | |
| Asymptomatic VTE | 0 | 0 [0-2.7] | 0 | 0 [0-5.3] | 0 | 0 [0-5.3] |
| **Postoperative complications** | | | | | | |
| Any postoperative complication | 13 | 9.4 [5.6-15.5] | 5 | 7.3 [3.1-15.9] | 8 | 11.6 [6.0-21.2] |
| Superficial SSI | 2 | 1.4 [0.4-5.1] | 1 | 1.4 [0.1-7.8] | 1 | 1.4 [0.1-7.8] |
| Deep SSI | 1 | 0.7 [0.0-4.0] | 0 | 0 [0-5.3] | 1 | 1.4 [0.1-7.8] |
| Organ-space SSI | 4 | 2.9 [1.1-7.2] | 1 | 1.4 [0.1-7.8] | 3 | 4.3 [1.5-12.0] |
| Wound dehiscence | 2 | 1.4 [0.4-5.1] | 2 | 2.9 [0.8-10.0] | 0 | 0 [0-5.3] |
| Deep venous thrombosis | 0 | 0 [0-2.7] | 0 | 0 [0-5.3] | 0 | 0 [0-5.3] |
| Urinary tract infection | 1 | 0.7 [0.0-4.0] | 0 | 0 [0-5.3] | 1 | 1.4 [0.1-7.8] |
| Readmission | 1 | 0.7 [0.0-4.0] | 0 | 0 [0-5.3] | 1 | 1.4 [0.1-7.8] |
| Reoperation | 0 | 0 [0-2.7] | 0 | 0 [0-5.3] | 0 | 0 [0-5.3] |
| Other | 2 | 1.4 [0.4-5.1] | 1 | 1.4 [0.1-7.8] | 1 | 1.4 [0.1-7.8] |
| Dindo-Clavien classification | | | | | | |
| Grade I | 5 | 3.6 [1.6-8.2] | 4 | 5.8 [2.3-14.0] | 1 | 1.5 [0.1-7.8] |
| Grade II | 5 | 3.6 [1.6-8.2] | 1 | 1.4 [0.1-7.8] | 4 | 5.8 [2.3-14.0] |
| Grade IIIa | 3 | 2.2 [0.7-6.2] | 0 | 0 [0-5.3] | 3 | 4.3 [1.5-12.0] |
| Grade IIIb | 0 | 0 [0-2.7] | 0 | 0 [0-5.3] | 0 | 0 [0-5.3] |
| Allergic reaction | 6 | 4.3 [2.0-9.2] | 3 | 4.3 [1.5-12.0] | 3 | 4.3 [1.5-12.0] |
| **SG** | n = 114 | | n = 55 | | n = 59 | |
| Asymptomatic VTE | 1 | 0.9 [0.0-4.8] | 0 | 0 [0-6.5] | 1 | 1.7 [0.1-9.0] |
| **Postoperative complications** | | | | | | |
| Any postoperative complication | 10 | 8.8 [4.8-15.4] | 3 | 5.5 [1.9-14.9] | 7 | 11.9 [5.9-22.5] |
| Superficial SSI | 0 | 0 [0-3.3] | 0 | 0 [0-6.5] | 0 | 0 [0-6.1] |
| Deep SSI | 0 | 0 [0-3.3] | 0 | 0 [0-6.5] | 0 | 0 [0-6.1] |
| Organ-space SSI | 1 | 0.9 [0.0-4.8] | 1 | 1.818 [0.1-9.6] | 0 | 0 [0-6.1] |
| Wound dehiscence | 1 | 0.9 [0.0-4.8] | 0 | 0 [0-6.5] | 1 | 1.7 [0.1-9.0] |
| Deep venous thrombosis | 1 | 0.9 [0.0-4.8] | 0 | 0 [0-6.5] | 1 | 1.7 [0.1-9.0] |
| Urinary tract infection | 1 | 0.9 [0.0-4.8] | 0 | 0 [0-6.5] | 1 | 1.7 [0.1-9.0] |
| Readmission | 3 | 2.6 [0.9-7.5] | 1 | 1.8 [0.1-9.6] | 2 | 3.4 [0.9-11.5] |
| Reoperation | 0 | 0 [0-3.3] | 0 | 0 [0-6.5] | 0 | 0 [0-6.1] |
| Other | 3 | 2.6 [0.9-7.5] | 1 | 1.8 [0.1-9.6] | 2 | 3.4 [0.9-11.5] |
| Dindo-Clavien classification | | | | | | |
| Grade I | 3 | 2.6 [0.9-7.5] | 0 | 0 [0-6.5] | 3 | 5.1 [1.7-13.9] |
| Grade II | 6 | 5.3 [2.4-11.0] | 2 | 3.6 [1.0-12.3] | 4 | 6.8 [2.7-16.2] |
| Grade IIIa | 1 | 0.9 [0.0-4.8] | 1 | 1.818 [0.1-9.6] | 0 | 0 [0-6.1] |
| Grade IIIb | 0 | 0 [0-3.3] | 0 | 0 [0-6.5] | 0 | 0 [0-6.1] |
| Allergic reaction | 0 | 0 [0-3.3] | 0 | 0 [0-6.5] | 0 | 0 [0-6.1] |
| **RS** | n = 17 | | n = 10 | | n = 7 | |
| Asymptomatic VTE | 0 | 0.0 [0.0-18.4] | 0 | 0.0 [0.0-27.8] | 0 | 0.0 [0.0-35.4] |
| **Postoperative complications** | | | | | | |
| Any postoperative complication | 3 | 17.6 [6.2-41.0] | 1 | 10.0 [0.5-40.4] | 2 | 28.6 [8.2-64.1] |

| | | | | | | |
|---|---|---|---|---|---|---|
| Superficial SSI | 0 | 0.0 [0.0-18.4] | 0 | 0.0 [0.0-27.8] | 0 | 0.0 [0.0-35.4] |
| Deep SSI | 0 | 0.0 [0.0-18.4] | 0 | 0.0 [0.0-27.8] | 0 | 0.0 [0.0-35.4] |
| Organ-space SSI | 0 | 0.0 [0.0-18.4] | 0 | 0.0 [0.0-27.8] | 0 | 0.0 [0.0-35.4] |
| Wound dehiscence | 0 | 0.0 [0.0-18.4] | 0 | 0.0 [0.0-27.8] | 0 | 0.0 [0.0-35.4] |
| Deep venous thrombosis | 0 | 0.0 [0.0-18.4] | 0 | 0.0 [0.0-27.8] | 0 | 0.0 [0.0-35.4] |
| Urinary tract infection | 0 | 0.0 [0.0-18.4] | 0 | 0.0 [0.0-27.8] | 0 | 0.0 [0.0-35.4] |
| Readmission | 1 | 5.9 [0.3-27.0] | 0 | 0.0 [0.0-27.8] | 1 | 14.3 [0.7-51.3] |
| Reoperation | 1 | 5.9 [0.3-27.0] | 1 | 10.0 [0.5-40.4] | 0 | 0.0 [0.0-35.4] |
| Other | 1 | 5.9 [0.3-27.0] | 0 | 0.0 [0.0-27.8] | 1 | 14.3 [0.7-51.3] |
| Dindo-Clavien classification | | | | | | |
| Grade I | 1 | 5.9 [0.3-27.0] | 0 | 0.0 [0.0-27.8] | 1 | 14.3 [0.7-51.3] |
| Grade II | 0 | 0.0 [0.0-18.4] | 0 | 0.0 [0.0-27.8] | 0 | 0.0 [0.0-35.4] |
| Grade IIIa | 1 | 5.9 [0.3-27.0] | 0 | 0.0 [0.0-27.8] | 1 | 14.3 [0.7-51.3] |
| Grade IIIb | 1 | 5.9 [0.3-27.0] | 1 | 10.0 [0.5-40.4] | 0 | 0.0 [0.0-35.4] |
| Allergic reaction | 0 | 0.0 [0.0-18.4] | 0 | 0.0 [0.0-27.8] | 0 | 0.0 [0.0-35.4] |

VTE: venous thromboembolism; SSI: surgical site infection; CI: confidence interval; SG sleeve gastrectomy; RYGB: Roux-en-Y gastric bypass; RS: revisional surgery

© 2023 Kröll D et al. *JAMA Network Open.*

**eFigure 1: Cumulative event rate of symptomatic and asymptomatic VTE (iMTT data set, n=257)**

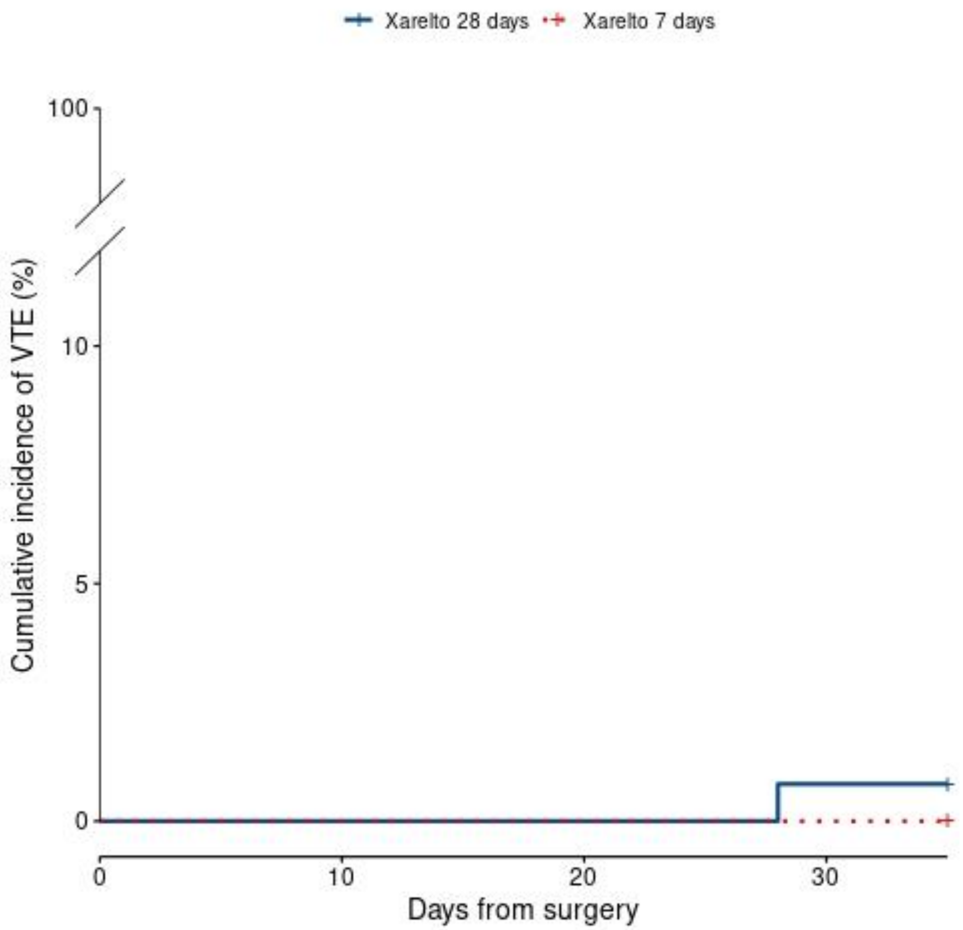


VTE: Venous thromboembolism

© 2023 Kröll D et al. *JAMA Network Open.*

**eFigure 2: Cumulative event rate of any major and clinically relevant non-major bleeding (safety population, n=269)**

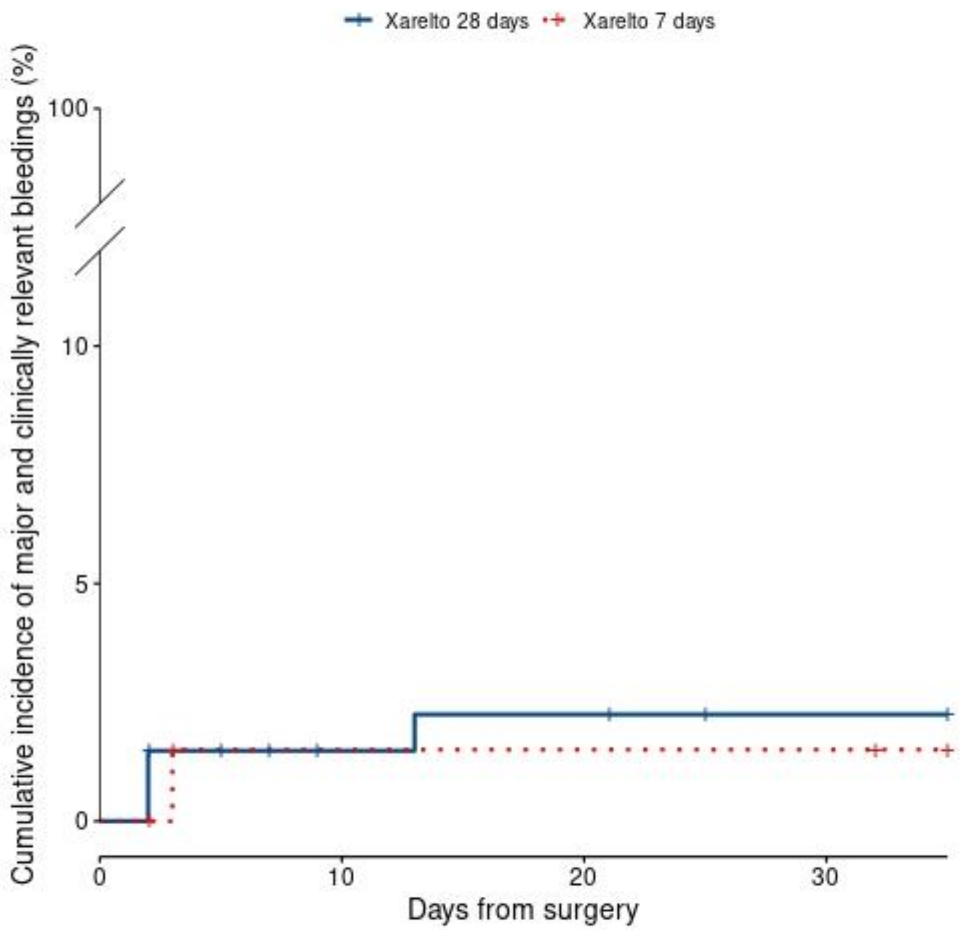


© 2023 Kröll D et al. *JAMA Network Open.*
