# Supplementary material for: Efficacy and Safety of Rivaroxaban for Postoperative Thromboprophylaxis in Patients After Bariatric Surgery: A Randomized Clinical Trial
Source: JAMA Netw Open. 2023 May 25;6(5):e2315241. doi: 10.1001/jamanetworkopen.2023.15241 (PMC10214035; doi:10.1001/jamanetworkopen.2023.15241)
Supplement: Supplement 3. — Data Sharing Statement [file jamanetwopen-e2315241-s003.pdf]

Data Sharing Statement

Kröll. Efficacy and Safety of Rivaroxaban for Postoperative Thromboprophylaxis in Patients After Bariatric Surgery. *JAMA Netw Open*. Published May 25, 2023.
doi:10.1001/jamanetworkopen.2023.15241

Data
Data available: No

Additional Information
Explanation for why data not available: Data can be requested and will be provided following review and approval of a research proposal and Statistical Analysis Plan (SAP) and execution of a Data Sharing Agreement (DSA).
